# From promoter motif to cardiac function: a single DPE motif affects transcription regulation and organ function *in vivo*

**DOI:** 10.1242/dev.202355

**Published:** 2024-07-29

**Authors:** Anna Sloutskin, Dekel Itzhak, Georg Vogler, Hadar Pozeilov, Diana Ideses, Hadar Alter, Orit Adato, Hadar Shachar, Tirza Doniger, Galit Shohat-Ophir, Manfred Frasch, Rolf Bodmer, Sascha H. Duttke, Tamar Juven-Gershon

**Affiliations:** ^1^ The Mina and Everard Goodman Faculty of Life Sciences, Bar-Ilan University, Ramat Gan 5290002, Israel; ^2^ Development, Aging and Regeneration Program, Sanford Burnham Prebys Medical Discovery Institute, La Jolla, CA 92037, USA; ^3^ Division of Developmental Biology, Department of Biology, Friedrich-Alexander University of Erlangen-Nürnberg, Erlangen 91058, Germany; ^4^ School of Molecular Biosciences, College of Veterinary Medicine, Washington State University, Pullman, WA 99164, USA

**Keywords:** RNA Polymerase II transcription, Core promoter, Gene expression, Nascent transcription, *Drosophila*, Heart development

## Abstract

Transcription initiates at the core promoter, which contains distinct core promoter elements. Here, we highlight the complexity of transcriptional regulation by outlining the effect of core promoter-dependent regulation on embryonic development and the proper function of an organism. We demonstrate *in vivo* the importance of the downstream core promoter element (DPE) in complex heart formation in *Drosophila*. Pioneering a novel approach using both CRISPR and nascent transcriptomics, we show the effects of mutating a single core promoter element within the natural context. Specifically, we targeted the downstream core promoter element (DPE) of the endogenous *tin* gene, encoding the Tinman transcription factor, a homologue of human NKX2-5 associated with congenital heart diseases. The 7 bp substitution mutation results in massive perturbation of the Tinman regulatory network that orchestrates dorsal musculature, which is manifested as physiological and anatomical changes in the cardiac system, impaired specific activity features, and significantly compromised viability of adult flies. Thus, a single motif can have a critical impact on embryogenesis and, in the case of DPE, functional heart formation.

## Teaser

**Summary:** A single DPE core promoter motif in *tinman* is crucial for developmental gene regulatory networks *in vivo*, and for adult heart function and viability.

## INTRODUCTION

Transcription initiation by RNA Polymerase II (Pol II) occurs at the core promoter region (–40 to +40 relative to the transcription start site; TSS) (Heintzman and Ren, 2007; Juven-Gershon et al., 2008b). Once regarded as a universal component whose mechanism of action is shared by all protein-coding genes, it is nowadays appreciated that core promoters are divergent in their composition and function. Interestingly, distinct core promoter compositions have been demonstrated to result in various transcriptional outputs, and to be associated with specific gene regulatory networks (Danino et al., 2015; Haberle and Stark, 2018; Sloutskin et al., 2021; Vo Ngoc et al., 2019).

Core promoters may contain one or more short DNA sequence motifs, termed core promoter elements or motifs that confer specific properties to the core promoter (Anish et al., 2009; Burke and Kadonaga, 1996, 1997; Deng and Roberts, 2005; Goldberg, 1979; Hendrix et al., 2008; Kutach and Kadonaga, 2000; Lagrange et al., 1998; Lim et al., 2004; Lo and Smale, 1996; Ohler et al., 2002; Parry et al., 2010; Smale and Baltimore, 1989; Theisen et al., 2010; Tokusumi et al., 2007; Vo Ngoc et al., 2017, 2020; Wang et al., 2017). One such motif is the downstream core promoter element (DPE), which is enriched in the promoters of developmentally regulated genes, including most homeotic (Hox) genes (Juven-Gershon et al., 2008a) and those regulating dorsal-ventral patterning (Zehavi et al., 2014a,b). Interestingly, the DPE is found in the promoters of many genes involved in heart and mesodermal development (Sloutskin et al., 2015), including *tinman* (*tin*), which is the *Drosophila* homologue of the human gene *NKX2-5*. *tin* encodes an extensively studied transcription factor that orchestrates the formation of the heart and its associated tissues during *Drosophila* embryonic development (Azpiazu and Frasch, 1993; Bodmer, 1993). It has further been shown to play a key role in early mesoderm patterning and in the formation of all dorsal mesodermal derivatives, which, in addition to working cardioblasts, valve cardioblasts and pericardial cells, include visceral and specific somatic muscles (Bryantsev and Cripps, 2009; Cripps and Olson, 2002; Reim and Frasch, 2010; Rotstein and Paululat, 2016; Zaffran et al., 2006).

It has previously been shown that introducing substitution mutations in the DPE of the *tin* core promoter significantly reduces transcriptional output in reporter transfection assays and *in vitro* transcription analysis with embryonic extracts (Zehavi et al., 2014a). However, in the genome, mutations in regulatory elements are often buffered, in part due to a diversity of sequence-specific transcription factors and the functional redundancy of regulatory motifs (Jin et al., 2013; Osterwalder et al., 2018; Spivakov, 2014). This increases the need to elucidate the role of DPE in the whole organism*.* To this end, we mutated the DPE motif of the *tin* core promoter (*tin^mDPE^*) using a CRISPR-based strategy (Levi et al., 2020).

Our findings indicate that mutation of the DPE motif is sufficient to reduce *tin* expression, at both the RNA and protein levels, with no accompanied changes detected in *tin* expression patterns. Although the dorsal vessel is formed in *tin^mDPE^* homozygous embryos, both alleles are required for survival, with one copy of *tin^mDPE^* unable to fully compensate for a loss-of-function *tinman* allele *in trans*. Importantly, major defects in adult heart physiology, anatomy and distinct motoric features were observed. Nascent transcription analysis of *tin^WT^* and *tin^mDPE^* homozygous embryos detected differential expression of *tin* target genes, many of which are implicated in heart development and tube formation. Moreover, DPE-like motifs are significantly enriched among the differentially regulated peaks.

Altogether, our results demonstrate the feasibility and importance of studying core promoter elements in their native genomic context, underline the function of the DPE motif of *tin* in dorsal vessel specification in *Drosophila*, and highlight the importance a single core promoter element can have in development, viability and functional heart formation.

## RESULTS

### Reduced expression levels of endogenous *tinman* in mDPE strains

**Fig. 1.**
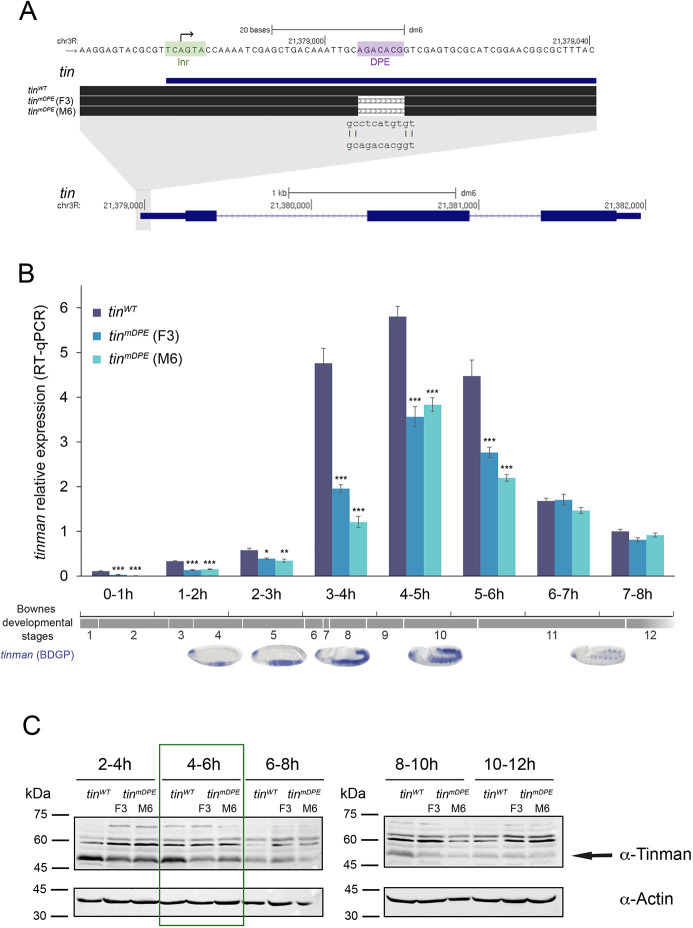
**Endogenous *tinman* RNA and protein levels are reduced in *tin^mDPE^ Drosophila melanogaster* embryos in distinct developmental time intervals.** (A) Summary of the *tinman* core promoter and gene. The Inr and downstream core promoter element (DPE) motifs are annotated, along with the motif sequences in the generated flies: *tin^WT^*, and *tin^mDPE^*- F3 and M6 strains. The top and bottom sequences are at different scales. (B) *tin^WT^* and *tin^mDPE^* (F3 and M6) embryos were collected at 1 h intervals for the first 8 h of development, and their RNA was purified and reverse transcribed. Endogenous *tinman* expression levels were measured by RT-qPCR and analyzed using the StepOnePlus software. Each qPCR experiment was performed in triplicate. Error bars represent 95% confidence interval, *n*≥3 for each time interval. *P<0.05, **P<0.01, ***P<0.001 (one-way ANOVA followed by Tukey's post-hoc-test). Only comparisons with the *tin^WT^* samples are presented. Bownes developmental stages (Interactive Fly) and the expected *tinman* expression patterns are indicated below the relevant time intervals (*in situ* hybridization patterns reproduced, with permission, from Berkeley *Drosophila* Genome Project (https://insitu.fruitfly.org/cgi-bin/ex/insitu.pl) (Tomancak et al., 2007). (C) Tinman protein levels are significantly lower in 4-6 h *tin^mDPE^* embryos compared with *tin^WT^.* Representative western blot images of protein extracts from embryos collected at 2-4 h, 4-6 h, 6-8 h, 8-10 h or 10-12 h time intervals. For each membrane, embryos from the same fly populations were collected. Western blotting of each membrane was initially performed using rabbit anti-Tinman antibodies. The levels of Actin as a loading control were detected using mouse anti-Actin antibodies.

To investigate the contribution of DPE to the regulation of the *tin* gene *in vivo*, we used the co-CRISPR approach to substitute the endogenous DPE sequence (AGACACG) with the non-functional CTCATGT (Levi et al., 2020) (Fig. 1A). Two independent *tin^mDPE^ Drosophila melanogaster* strains, namely F3 and M6, were extensively characterized in this study, and compared with the injected strain, Cas9, which is referred to herein as *tin^WT^*.

Quantification of endogenous *tin* RNA levels in *tin^WT^* and *tin^mDPE^* embryos within 1 h windows during the first 8 h of embryonic development (up to Bownes developmental stage 12 embryos, Fig. 1B) revealed a marked reduction in endogenous *tin* expression levels in *tin^mDPE^* embryos. Differences in *tin* expression levels were evident, starting from the earliest tested time interval (0-1 h), and were most substantial at 3-4 h, when Tinman activity is crucial for mesoderm development (Yin et al., 1997; Zaffran et al., 2006). Later in development (6-7 h and 7-8 h, stages 11-12), *tin* levels were indistinguishable between *tin^mDPE^* and *tin^WT^* embryos. Despite differences in *tin* expression levels at 0-6 h, no apparent difference was detected in the *tin* expression pattern in early *tin^mDPE^* and *tin^WT^* embryos by *in situ* hybridization (Fig. S1). Both *tin^mDPE^* strains and *tin^WT^* exhibited a *tin* expression pattern that highly matched that of the reported expression (Azpiazu and Frasch, 1993; Bodmer, 1993; Bodmer et al., 1990). Notably, Tinman protein levels were significantly lower in 4-6 h *tin^mDPE^* embryos compared with *tin^WT^* (Fig. 1C, Fig. S2, Table S1). Thus, mutating endogenous *tin* DPE results in reduced *tin* RNA and protein expression levels in distinct developmental time intervals.

### Functional effects of reduced Tinman expression

**Fig. 2.**
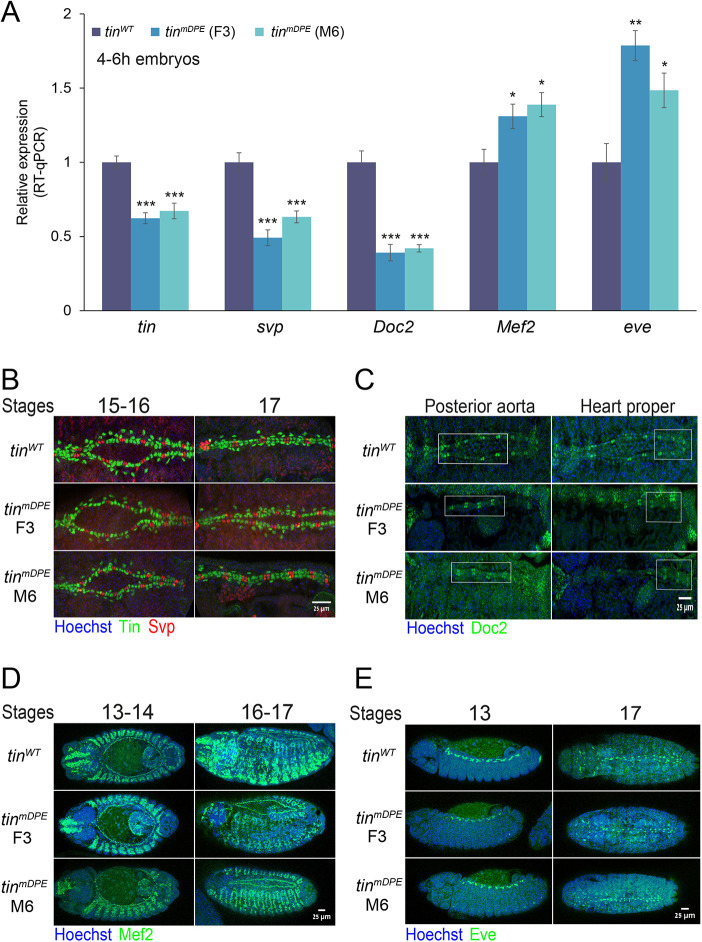
**The expression of distinct Tinman target genes is affected in early *tin^mDPE^* embryos, yet dorsal vessel formation, as evident from Svp, Doc2, Mef2 and Eve protein expression patterns at later stages, appears intact in *tin^mDPE^* embryos.** (A) Endogenous *tin*, *svp*, *Doc2*, *Mef2* and *eve* RNA levels were quantified in 4-6 h embryos (Bownes stage ∼10) using RT-qPCR and analyzed using the StepOnePlus software. Expression of all genes in *tin^WT^* is defined to be 1. Each qPCR experiment was performed in triplicate. Error bars represent 95% confidence interval. *P<0.05, **P<0.01, ***P<0.001 (one-way ANOVA followed by Tukey's post-hoc test). Only comparisons with *tin^WT^* samples are presented, *n*≥3 (*n*=number of biological replicates, each tested in three technical replicates). (B-E) *tin^WT^* and *tin^mDPE^* (F3 and M6) embryos were stained using anti-Tin (green) and anti-Svp (red) (B), anti-Doc2 (green) (C), anti-Mef2 (green) (D), and anti-Eve (green) (E) antibodies, and counterstained with a nuclear dye (Hoechst, blue). Representative embryos (stages ≥13) are shown for each fly line. *Z*-stack 3D projections are shown. Anterior is towards the left. Scale bars: 25 µm.

*tinman* encodes a homeodomain transcription factor that is a master regulator of mesoderm and heart development (Bryantsev and Cripps, 2009). We therefore tested the endogenous expression levels of Tinman and its target genes *seven up* (*svp*), *Dorsocross 2* (*Doc2*), *Myocyte enhancer factor 2* (*Mef2*) and *even skipped* (*eve*), as well as *tin* expression, in 4-6 h *tin^WT^* and *tin^mDPE^* embryos (stages 8-10) (Fig. 2A). Both *svp* and *Doc2*, two genes involved in heart development (Lo and Frasch, 2003; Reim and Frasch, 2005; Ryan et al., 2007) were downregulated in both *tin^mDPE^* strains at the 4-6 h time interval. The levels of *Mef2* and *eve*, which have previously been shown to be perturbed in classical *tin* knockouts (Azpiazu and Frasch, 1993; Bodmer, 1993; Gajewski et al., 1997), were slightly elevated in early *tin^mDPE^* embryos (stages 8-10) (Fig. 2A). Nevertheless, the formation of an apparently normal dorsal vessel in late *tin^mDPE^* embryos (stage ≥13) was evident, based on either Tin, Svp, Doc2, Mef2 or Eve protein localization (Fig. 2B-E).

Misexpression of Odd-skipped (Odd), a key marker of pericardial cells (Ward and Skeath, 2000), in *tin^mDPE^* embryos was detected, presenting an ectopic Odd pattern almost completely masking the Odd-positive pericardial cells (Fig. S3). Nevertheless, based on Tin and Odd staining patterns (Fig. S3), as well as on Svp, Doc2, Mef2 and Eve staining patterns (Fig. 2B-E), proper dorsal vessel formation is evident in both homozygous *tin^mDPE^* embryos.

### A single *tin^mDPE^* allele is unable to restore viability and dorsal vessel structure upon *tin* loss

**Fig. 3.**
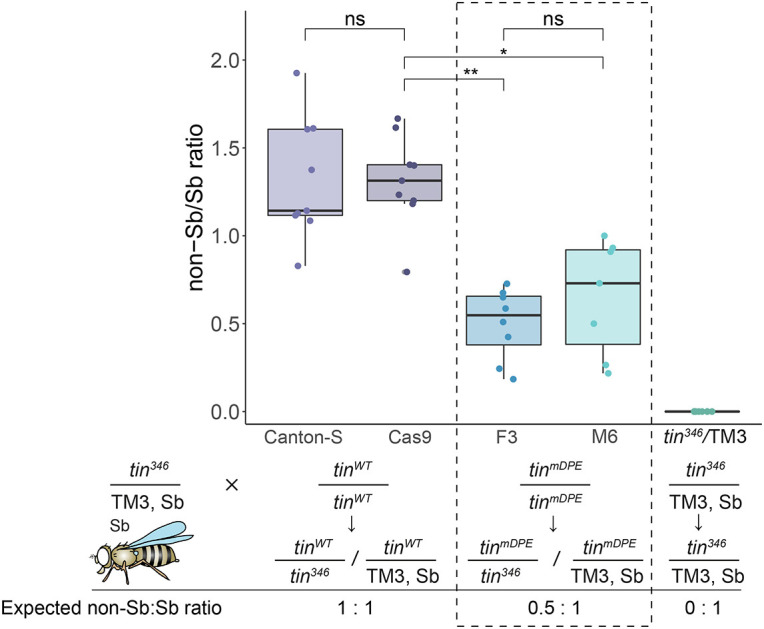
**Mutation of *tinman* endogenous downstream core promoter element (DPE) reduces the viability of mature flies when tested *in trans* to *tin* null mutation.** *tin^WT^* (Canton-S, Cas9) and *tin^mDPE^* (F3 and M6) strains were each crossed to *tin^346^*/TM3, Sb flies. The Sb phenotype of eclosed flies was used to distinguish between *tin^mDPE^* over *tin^346^* (null) or the wild-type allele; *tin^346^* was also scored as a background. *P<0.05, **P<0.01 (one-way nested ANOVA followed by Tukey's HSD post-hoc test). Schematic representation of the relevant cross and the expected non-Sb/Sb ratio is indicated. Schematic fly image is from Roote and Prokop (2017) (see also Roote and Prokop, 2013). The data and genotypes of the *tin^mDPE^* flies that were crossed with the *tin* null heterozygote are outlined. Boxes indicate interquartile range (IQR) and median, whiskers indicate the range within 1.5 times the IQR from the quartiles, excluding outliers.

Unlike *tin* null homozygous flies, which are not viable (Azpiazu and Frasch, 1993), *tin^mDPE^* homozygous flies were viable but appeared frail. To examine whether a single *tin^mDPE^* allele can compensate for the loss of *tin*, we crossed either *tin^WT^* or *tin^mDPE^* flies with flies carrying a *tin* null allele maintained over a balancer (*tin^346^*/[TM3, *eve*-LacZ]) (Fig. S4). Eclosed flies were scored for the Stubble (Sb) phenotype (indicative of the TM3 balancer), which enables the distinction between the examined allele over a *tin^346^* (non-Sb) or over a *tin* wild-type (balancer, Sb) allele. Thus, a non-Sb/Sb ratio reflects the presence of a *tin^WT^* or *tin^mDPE^* allele *in trans* to a *tin* null allele. For full compensation by the *tin* wild-type allele, we expect the non-Sb/Sb ratio to be equal to 1, i.e. the same number of rescued *tin^346^*/*tin^WT^* flies and *tin^346^*/TM3 flies. Strikingly, only half the number of viable flies were observed in *tin^mDPE^/tin^346^* when compared with *tin^WT^/tin^346^* (Fig. 3). These data indicate a substantial decrease in viability when *tin^mDPE^* is present as a single copy, in contrast to a single copy of the *tin* wild-type allele when tested *in trans* to a null allele. These results strongly support the notion that the decreased mRNA expression levels lead to a reduced function of *tin^mDPE^*.

**Fig. 4.**
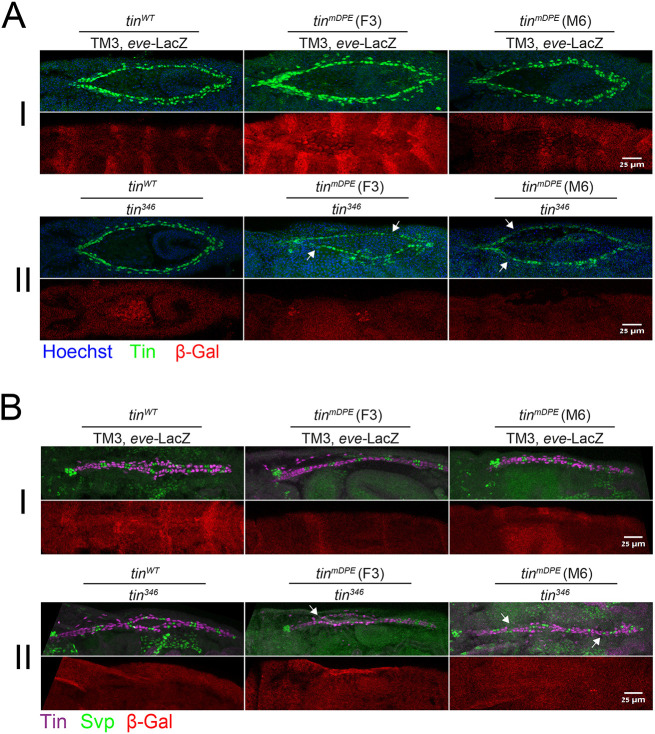
**The expression patterns of Tinman and Svp in cardioblasts are aberrant in *tin^mDPE^* embryos when tested *in trans* to the *tin* null mutation.** *tin^WT^* and *tin^mDPE^* (F3 and M6) strains were crossed to the *tin^346^*/[TM3, *eve*-LacZ] strain. Embryos resulting from each cross were co-stained for β-Gal to identify β-Gal-positive embryos, i.e. *tin^WT^*/[TM3, *eve*-LacZ] and *tin^mDPE^*/[TM3, *eve*-LacZ] (I), and β-Gal-negative embryos, i.e. *tin^WT^*/*tin^346^* and *tin^mDPE^*/*tin^346^* (II). (A) Embryos from all three crosses were stained using anti-Tin (green) and anti-β-Gal (red) antibodies, and counterstained with a nuclear dye (Hoechst, blue). Two representative embryos (stages ∼13-15) are shown for each fly line. Arrows indicate the absence of Tin-positive cells in β-Gal-negative embryos. (B) Embryos from all three crosses were stained using anti-Svp (green), anti-Tin (purple) and anti-β-Gal (red) antibodies. Two representative embryos (stages ∼16-17) are shown for each fly line. Arrows indicate the absence of Svp-positive cells in β-Gal-negative embryos. *Z*-stack 3D projections are shown. Anterior is towards the left. Scale bars: 25 µm.

To determine whether Tin protein expression pattern and heart development are impaired in *tin^mDPE^* embryos when tested *in trans* to the *tin* null mutation, *tin^WT^* and *tin^mDPE^* (F3 and M6) strains were each crossed to the *tin^346^*/[TM3, *eve*-LacZ] strain (Fig. S4). Embryos resulting from each cross were co-stained for Tin and β-Gal to identify β-Gal-negative embryos, i.e. *tin^WT^*/*tin^346^* and *tin^mDPE^*/*tin^346^*. Remarkably, the expression pattern of Tin shows frequent gaps in cardioblast rows in *tin^mDPE^* embryos when tested *in trans* to the *tin* null mutation (Fig. 4A). Svp has previously been shown to be expressed in 14 cells distributed as seven pairs along the vessel in cells where Tinman is not expressed (Lo and Frasch, 2001; Zaffran et al., 2006). Notably, the Svp expression pattern seems to differ between *tin^WT^*/*tin^346^* and *tin^mDPE^*/*tin^346^* embryos, with some segments in *tin^mDPE^*/*tin^346^* having only one of two Svp-positive cells (Fig. 4B).

In addition to its function in heart formation, Tinman is required for dorsal somatic and visceral muscle formation (Azpiazu and Frasch, 1993; Bodmer, 1993). To examine whether the reduced viability of *tin^mDPE^/tin^346^* flies could result from defects in non-cardiac muscle formation, we stained embryos resulting from crosses of *tin^WT^* and *tin^mDPE^* (F3 and M6) strains to *tin^346^*/[TM3, *eve*-LacZ] with anti-β3Tubulin antibodies for somatic musculature, and anti-Optomotor blind-related gene 1 (Org1) antibodies for visceral mesoderm (Fig. S5). Both β3Tubulin and Org1 expression were similar between *tin^WT^*/*tin^346^* and *tin^mDPE^*/*tin^346^* embryos, indicating that the reduced viability of *tin^mDPE^*/*tin^346^* does not result from impaired somatic and/or visceral muscle function.

**Fig. 5.**
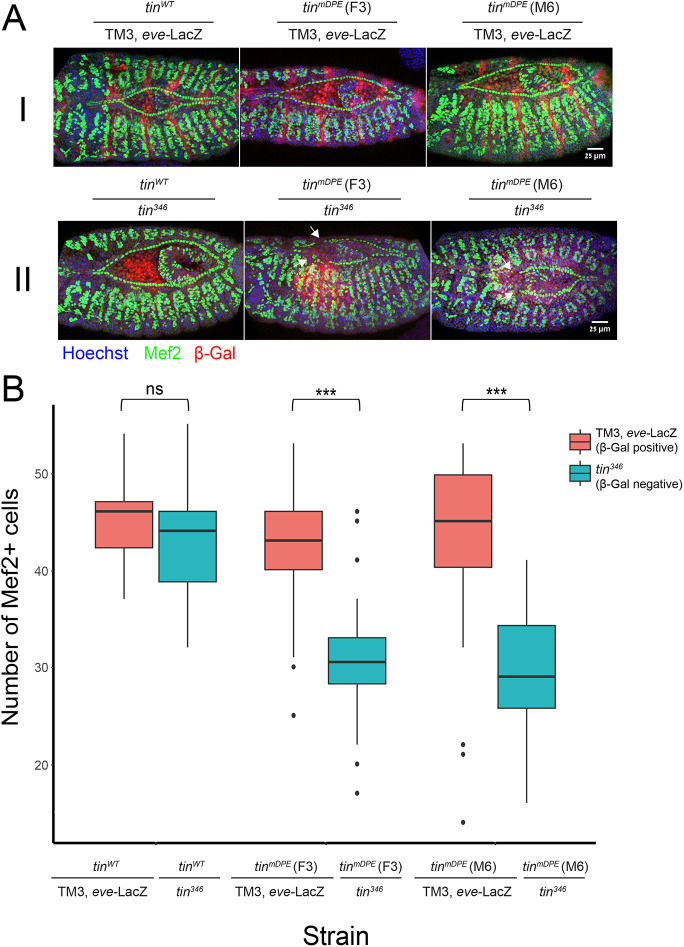
**The number of Mef2-positive cardioblasts is reduced in *tin^mDPE^* embryos when tested *in trans* to the *tin* null mutation.** *tin^WT^* and *tin^mDPE^* (F3, M6) strains were crossed to the *tin^346^*/[TM3, *eve*-LacZ] strain. Embryos resulting from each cross were co-stained for β-Gal to identify β-Gal-positive embryos, i.e. *tin^WT^*/[TM3, *eve*-LacZ] and *tin^mDPE^*/[TM3, *eve*-LacZ] (I), and β-Gal-negative embryos, i.e. *tin^WT^*/*tin^346^* and *tin^mDPE^*/*tin^346^* (II). (A) Embryos from all three crosses were stained using anti-Mef2 (green) and anti-β-Gal (red) antibodies, and counterstained with a nuclear dye (Hoechst, blue). Two representative embryos (stages ∼14-16) are shown for each fly line. *Z*-stack 3D projections are shown. Anterior is towards the left. Scale bars: 25 µm. Arrows indicate the absence of Mef2-positive cells within the β-Gal-negative embryos. (B) Mef2-positive (Mef2+) cells were counted in embryos resulting from each of the crosses. The β-Gal staining phenotype of embryos was used to distinguish between *tin^mDPE^* over *tin^346^* (null) or the wild-type allele (TM3, *eve*-LacZ). Wilcoxon tests were carried out (***P<0.001) followed by Bonferroni's correction for multiple testing, to assess the difference in the number of Mef2+ cells counted between the embryos that were β-Gal positive and β-Gal negative for each cross. Boxes indicate interquartile range (IQR) and median, whiskers indicate the range within 1.5 times the IQR from the quartiles, excluding outliers.

To further examine the effect of *tin* DPE on cardiac markers, we analyzed Mef2 protein expression in cardioblasts in embryos resulting from crosses of *tin^WT^* or *tin^mDPE^* (F3 and M6) strains to the *tin^346^*/[TM3, *eve*-LacZ] strain (Fig. 5). Remarkably, the number of Mef2-positive cardioblasts is significantly reduced in *tin^mDPE^* embryos when tested *in trans* to the *tin* null mutation (Fig. 5). Thus, although viable, phenotypic impacts on heart formation were evident, demonstrating the importance of the DPE in regulating *tinman* expression and function.

### Cardiac function and distinct activity features are impaired in *tin^mDPE^* adult flies

**Fig. 6.**
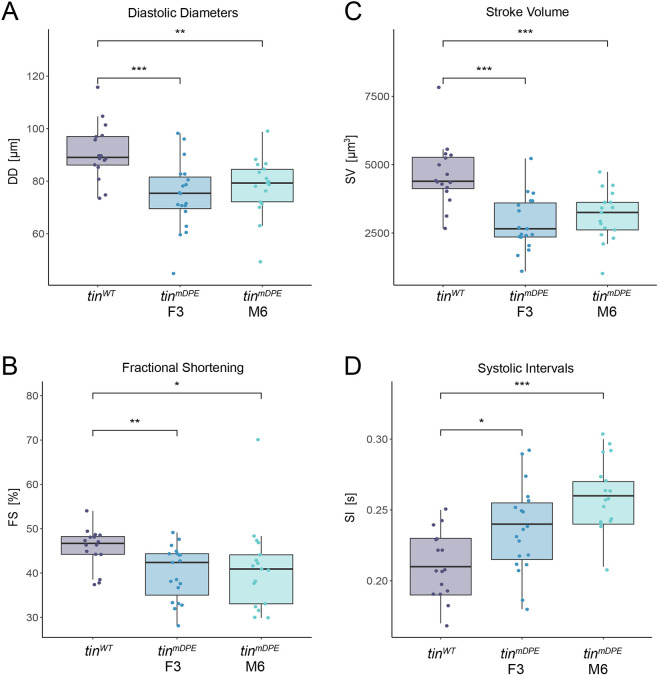
**Mutation of *tinman* endogenous downstream core promoter element (DPE) affects adult heart size and function.** (A-D) *tin^mDPE^* hearts (F3 and M6) have smaller diastolic diameters (DD; A) and reduced fractional shortening (FS; B), resulting in reduced stroke volume (SV; C). *Tin* DPE mutant hearts also exhibit prolonged systolic intervals (SI; D). In all panels, significance was tested using Wilcoxon tests (***P<0.001, **P<0.01, *P<0.05). Boxes indicate interquartile range (IQR) and median, whiskers indicate the range within 1.5 times the IQR from the quartiles, excluding outliers.

**Fig. 7.**
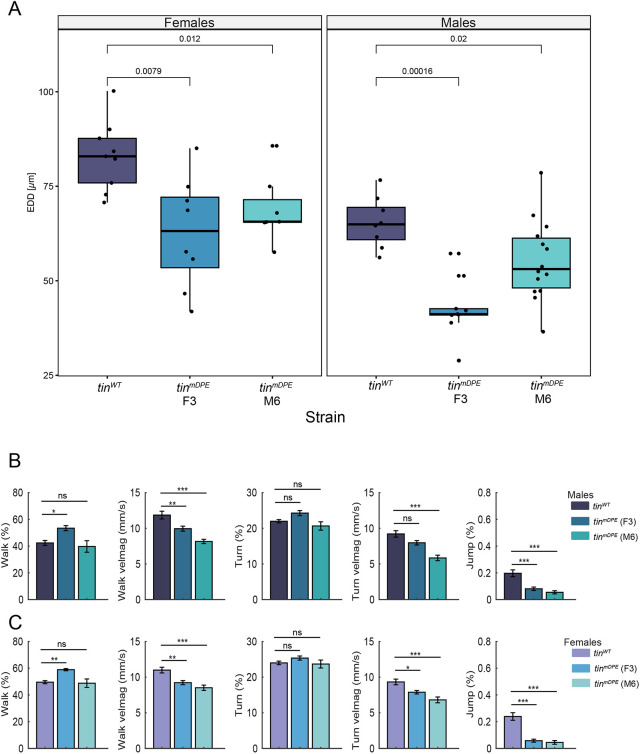
**Mutation of *tinman* downstream core promoter element (DPE) results in altered anatomy and distinct activity features.** (A) Cardiac diameters obtained from fixed heart tissue of *tin^WT^* and *tin^mDPE^* hearts (F3 and M6) have smaller diameters in both females and males. Adult hearts were dissected, fixed and stained to determine heart size and structure using anti-α-Spectrin antibodies and Alexa Fluor 633 phalloidin. For heart diameters, the distance between heart walls was measured in segment A2 posterior to the ostia cells. Statistical analysis was carried out using Wilcoxon tests, and graphs were created using R. Only comparisons to *tin^WT^* samples are presented. Boxes indicate interquartile range (IQR) and median, whiskers indicate the range within 1.5 times the IQR from the quartiles, excluding outliers. (B,C) The locomotor activity of 21-day-old *tin^mDPE^* lines F3 (blue), M6 (turquoise) and the *tin^WT^* line (purple) was measured using the FlyBowl system. The average percentage of time flies spent walking, changing orientation (turning) and jumping, and their average velocity during walking and turning are depicted for males (B) and females (C). *n*=9 (10 flies/arena). One-way ANOVA followed by Tukey's post-hoc test (*P<0.05, **P<0.01, ***P<0.001). Error bars indicate s.e.m. Only comparisons with the *tin^WT^* samples are presented.

We next tested whether *tin* DPE is necessary for heart function by analyzing adult homozygous *tin^WT^* and *tin^mDPE^* hearts using semi-automated heart analysis (SOHA; Fink et al., 2009). Adult wild-type and mDPE females were dissected, and their hearts were imaged using high-speed video recording, followed by a determination of spatial and temporal parameters [e.g. heart diameter during diastole (DD), systolic interval (SI) and stroke volume]. For both *tin^mDPE^* strains, we detected smaller diastolic diameters (Fig. 6A) and lower contractility (determined by fractional shortening, FS; Fig. 6B), resulting in a reduced stroke volume (Fig. 6C). Furthermore, *tin^mDPE^* mutant hearts showed longer systolic intervals (SIs; Fig. 6D), indicating prolonged contraction intervals. This suggests that the *tin* DPE is required to establish proper heart physiology. Notably, measurements of the diameters of fixed hearts in both 21-day-old female and male flies revealed significantly reduced end diastolic diameter (EDD) values in both *tin^mDPE^* strains (F3 and M6) compared with the *tin^WT^* strain (Fig. 7A), confirming the *in vivo* data. We did not observe differences in myofibrillar arrangement, in line with the hypomorphic character of the DPE mutants.

As heart parameters might directly influence viability, we assessed various locomotor features using the FlyBowl system, as described by Bentzur et al. (2021). Significantly reduced walking velocity (velmag) and average percentage jumping activity were observed in both 21-day-old adult male and female *tin^mDPE^* compared with *tin^WT^* flies (Fig. 7B,C). No significant changes in the average percentage of walking and turning activities or the turning velocity were observed between *tin^WT^* and both *tin^mDPE^* lines (Fig. 7B,C). In 4-day-old and 9-day-old male and female flies, the average percentage jumping activity was lower in both *tin^mDPE^* lines compared with the *tin^WT^* line (Fig. S6). Jumping activity declines with age, with a more pronounced reduction observed in *tin^WT^* flies. These findings suggest that impaired heart function, reduced walking velocity and decreased average percentage jumping activity may contribute to the reduced viability (Fig. 3).

### Nascent transcription analysis of *tin^mDPE^* embryos reveals significant changes in muscle and heart transcriptomes, preferentially among genes with DPE motifs

**Fig. 8.**
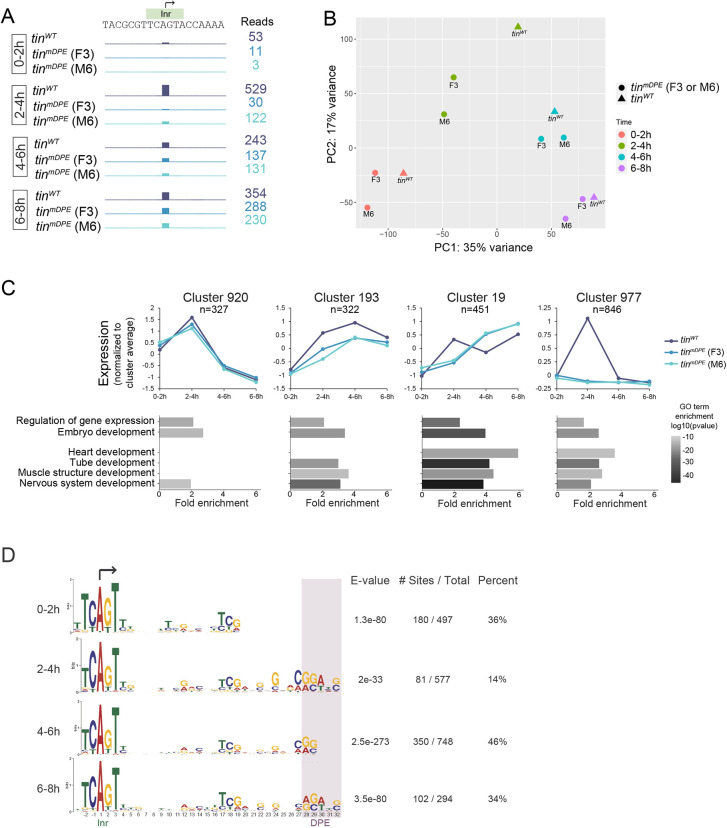
**Nascent transcription of developmental genes is altered in *tin^mDPE^* embryos.** csRNA-seq analysis was performed on 0-2 h, 2-4 h, 4-6 h and 6-8 h embryos for both *tin^WT^* and *tin^mDPE^* (F3 and M6) embryos. (A) Nascent transcription profile of the *tinman* locus, ±10 bp relative to the transcription start site. Scale is 0 to 530 for all tracks. (B) PCA analysis of the csRNAseq samples. Although samples are separated by developmental time, a difference between *tin^mDPE^* and *tin^WT^* strains is clearly evident for each time interval. (C) Expression and selected GO terms of representative clusters from HOMER's analyzeClusters.pl script. Top: mean expression of the cluster is shown for each strain at each time interval. Bottom: same selected GO terms are shown for each cluster, with the associated fold enrichment and *P*-value over genomic background. There is a strong association of cluster 19 with heart and tube development. See Fig. S10 and Table S2 for complete data. (D) A significant Inr+DPE resembling motif is evident for each tested time point following MEME analysis. Both raw numbers and percentages are presented. Inr and DPE motif locations are indicated; arrow indicates the transcription start site.

The reduced viability and functional heart parameters of *tin^mDPE^* flies indicated that this 7 bp substitution mutation within a single promoter results in major transcriptional changes. To quantify the proposed transcriptional changes genome-wide, we captured active or ‘nascent’ transcription, which offers a high-resolution analysis of impacted genomic loci and the underlying gene regulatory programs (Wissink et al., 2019). In addition, combining nascent assays with 5′cap selection enables the precise determination of the transcription initiation position and, consequently, the detection of any alternative initiation sites (Policastro and Zentner, 2021). Nuclei isolation is essential for many nascent methods (Wissink et al., 2019), but introduces bias during tissue (Lepage et al., 2021) or embryo digestion, whereas mechanical homogenization leads to the loss of delicate cells. To circumvent these challenges, we used capped small RNA sequencing (csRNA-seq), which, similar to GRO-cap, accurately captures nascent transcription start sites (TSSs) from total RNA (Duttke et al., 2019; Yao et al., 2022). csRNA-seq analysis of *tin^mDPE^* and *tin^WT^* embryos collected at 0-2 h, 2-4 h, 4-6 h and 6-8 h time intervals confirmed markedly reduced *tin* levels in *tin^mDPE^*, especially at 2-4 h (Fig. 8A). Interestingly, csRNA-seq analysis identified that transcription of *tin* in both the *tin^WT^* and *tin^mDPE^* lines initiates in a single TSS (Fig. 8A, Fig. S7), which is the same TSS as that depicted in Fig. 1A.

A principal component analysis (PCA) showed that samples first cluster by time and then by mDPE versus WT alleles (Fig. 8B). In accordance with the *tin* RT-qPCR results, the main overall difference between the mDPE and wild-type alleles is at the 2-4 h time interval. Within each time interval, the F3 and M6 samples clustered apart from the *tin^WT^* samples, indicative of impaired overall transcription and developmental programs. Notably, nascent RNA counts at 4-6 h of *tin* and its target genes *Doc2*, *Mef2* and *eve* (but not *svp*) (Fig. S8) are similar to their expression levels at 4-6 h analyzed by RT-qPCR (Fig. 2A).

csRNA-seq peaks were annotated based on the EPDnew database (Fig. S9), which identifies TSSs based on the 5′ cap of transcripts and quantifies the expression levels of individual transcripts based on experimental data (Meylan et al., 2020). The resulting clusters present either similar or differential *tin^mDPE^* and *tin^WT^* expression (Fig. S10, Table S2). The differential expression encompasses several modes, e.g. lower *tin^mDPE^* expression than *tin^WT^* expression, and alternating expression patterns (Fig. 8C, top, clusters #193 and #19, respectively). All the depicted clusters are similarly enriched for general GO terms, such as regulation of gene expression and embryo development, whereas differentially expressed clusters (193, 19 and 977) are specifically enriched for heart and muscle structure formation GO terms (Fig. 8C, bottom). Interestingly, nervous system development is also associated with the differentially expressed clusters.

In each time interval, differentially expressed csRNA-seq peaks were also enriched for Initiator (Inr) and DPE-like motifs (Fig. 8D). Given that the differentially expressed peaks for each time interval did not overlap (Fig. S11), the similarity of the over-represented motifs is striking. Although the exact motif composition and enrichment varies across the examined time intervals, combining the peaks that are differentially expressed in at least one time interval revealed a common DPE-like motif. This common motif is significantly enriched in all differentially expressed peaks (Fig. S12). As expected for a canonical DPE motif, all the DPE-like motifs are strictly positioned at the center of csRNA-seq peaks (Fig. S13). Notably, the enrichment of DPE in differentially expressed csRNA-seq peaks is in line with a significant enrichment of DPE in Tinman target genes previously identified by chromatin immunoprecipitation (ChIP) (Jin et al., 2013) (GEO deposit GSE41628) (Fig. S14).

Taken together, we demonstrate the *in vivo* importance of a single core promoter element, the DPE motif (Fig. S15). This 7 bp change within the *tinman* promoter sufficed to decrease Tinman levels, the result of which was marked changes in nascent transcription patterns, specifically of DPE-containing genes required for muscle and heart formation. Moreover, the insufficiency of a single *tin^mDPE^* copy to support viability in a deficient background highlights its crucial role in establishing the adequate Tinman levels required for functional heart formation.

## DISCUSSION

In this study, we present a detailed characterization of flies harboring a mutation in the endogenous sequence of a downstream core promoter element: the DPE motif. Mutating the DPE motif within the 5′ UTR of the *tin* gene resulted in reduced expression of both RNA and protein levels in the mDPE embryos (Fig. 1, Fig. S2), albeit with no apparent change in expression patterns detected using RNA *in situ* hybridization or antibody staining (Fig. 2B-E, Fig. S1). Interestingly, Svp, Doc2, Mef2 and Eve protein expression patterns are not affected in homozygous *tin^mDPE^* embryos, whereas the *Doc2* and *svp* RNA levels are markedly reduced in 4-6 h *tin^mDPE^* embryos (Fig. 2A). In *Drosophila* heart precursors, Tinman activates *svp* (Ryan et al., 2007), which encodes a repressor that acts through both DNA-binding competition and protein-protein interactions (Zelhof et al., 1995). *eve* expression in pericardial cells is *Doc2* independent (Reim and Frasch, 2005), suggesting that the reduction in Tinman levels after the DPE mutation differentially affects its target genes.

The reduced numbers of Tinman-positive cardioblasts (Fig. 4A), Svp-positive cardioblasts (Fig. 4B) and Mef2-positive cardioblasts (Fig. 5) in *tin^mDPE^* embryos when tested *in trans* to the *tin* null mutation indicate that the reduced *tin* activity in this genetic background leads to the specification of fewer cardioblasts. This reduction of cardioblast numbers is generally more pronounced in the anterior region of the embryonic heart tube (Figs 4, 5). Notably, the subdivision between anterior aorta lacking Svp expression and the more posterior heart regions that include Svp-expressing cells is regulated by Hox genes, which may contribute to the observed differential sensitivity to reduced *tin* activity along the anterior-posterior axis (Lo and Frasch, 2003). Although the majority of the Hox genes and mesodermal targets are DPE dependent (Juven-Gershon and Kadonaga, 2010; Sloutskin et al., 2021), it remains to be discovered whether Hox-driven patterning of the dorsal tube is mediated via the DPE. Although Tin is required for somatic and visceral muscle formation (Azpiazu and Frasch, 1993; Bodmer, 1993), dorsal somatic musculature and visceral musculature are unaffected, as predicted by the staining patterns of β3Tubulin and Org1, respectively (Fig. S5). Thus, heart development seems to be more dosage sensitive with respect to *tin* activity when compared with somatic musculature and visceral musculature.

Strikingly, both independent *tin^mDPE^* fly lines (F3 and M6) exhibit an overall decrease in adult heart function compared with the wild type, i.e. diminished diastolic diameters and decreased contractility, resulting in elongated contraction intervals and reduced stroke volume of *tin^mDPE^* homozygotes (Fig. 6). The functional effects manifested in the adult heart may result from reduced expression of *tin* and Tinman target genes required for normal heart physiology in the adult heart. The observed diminished heart function may also be due to subtle morphological alterations in the embryonic and/or adult heart, as detected in fixed adult hearts (Fig. 7A). As 21-day-old *tin^mDPE^* fly lines both have significantly reduced walking velocity and average percentage jumping activity, it is likely that the reduced viability of adult flies (Fig. 3) (as well as the reduced locomotor activity; Fig. 7B,C) results from the impaired heart function, through yet unknown mechanistic connections.

Developmental programs are largely executed by the transcription of the relevant genes. Capped-small RNA-seq (csRNA-seq) was developed in order to accurately quantify changes in transcription initiation during dynamic processes (Duttke et al., 2019). We applied csRNA-seq to study transcriptional dynamics at 2 h resolution, comparing *tin^WT^* and *tin^mDPE^* embryos from 0 to 8 h of development. Detected peaks were assigned to genes and clustered based on similar expression patterns. Reassuringly, development-related GO terms were enriched in most clusters, whereas heart-related and tube formation GO terms were associated with differentially regulated genes (Fig. 8C).

Remarkably, although homozygous *tin^mDPE^* flies are viable, they cannot fully compensate for the loss of *tin* (Fig. 3). Nevertheless, despite the reduced Tinman levels in the *tin^mDPE^* embryos, a dorsal vessel with a normal pattern of four Tinman-expressing cardioblasts is formed, presenting a very similar pattern to *tin* null heterozygotes. The viable phenotype of the *tin^mDPE^* flies strongly suggests the existence of a compensatory mechanism that ensures heart development is resistant to small-scale perturbations. In fact, several key mesodermal transcription factors, mainly Tinman, Pannier and Doc2, can bind the same genomic loci and define cardiac enhancers (Jin et al., 2013; Junion et al., 2012; Zinzen et al., 2009). Moreover, it has been suggested that functional flexibility exists, where the jointly bound transcription factors cooperate to recruit the relevant transcription factor to lower-affinity binding sites (Frasch, 2016). In such cases, it is more plausible that the target enhancer will be activated even in the presence of lower levels of the Tinman protein, due to perturbation of the *tin* DPE sequence motif. Consistent with the existence of a compensatory mechanism, not all Tinman target genes are affected by the reduction in Tinman levels in *tin^mDPE^* flies. Our findings provide further evidence for the complexity of the mesodermal regulatory network, which contains some redundant connections that are challenging to detect with standard genetic experiments. This redundancy may support the fine-tuning of expression circuits, which, in turn, generates a gene regulatory network that is more resistant to disruption. In this regard, the *tinman* DPE can be thought of as an element that fine-tunes *tinman* expression.

Compatibility between specific promoters and enhancers was demonstrated in different organisms (Butler and Kadonaga, 2001; Lim and Levine, 2021; Martinez-Ara et al., 2022; Zabidi et al., 2015). During development, *tin* is expressed in the head, trunk mesoderm, dorsal mesoderm and cardioblasts. Each expression pattern represents a specific developmental stage, and is explicitly controlled by a distinct enhancer integrating the relevant regulatory signals (Yin et al., 1997). These characterized enhancers were cloned and used to demonstrate that early Tinman expression is sufficient for dorsal vessel development and mesoderm specification (Zaffran et al., 2006). Remarkably, *tin* levels show the most pronounced decrease during the 3-4 h developmental phase (Fig. 1B), which is precisely when *twist* activates *tin*. These results suggest that the interaction between the *twist*-dependent intronic enhancer (Yin et al., 1997) and the *tin* promoter is highly sensitive to the presence of a functional DPE motif.

In *Drosophila* embryos, core promoter composition affects transcriptional dynamics profiles, detected with MS2-based reporters and a shared enhancer (Fukaya et al., 2016). Furthermore, the spacing of the enhancer-promoter pair modulates gene activity by changing the temporal and quantitative parameters of transcriptional bursts in the developing *Drosophila* embryo (Yokoshi et al., 2020). Direct examination of TATA-box and Inr elements using synthetic constructs in *Drosophila* revealed differences in the modes of action of these motifs (Pimmett et al., 2021). The essential role of native TATA box and DPE motifs within the *fushi tarazu* (*ftz*) promoter in transcriptional dynamics regulation was recently demonstrated *in vivo* (Yokoshi et al., 2022). Although the proper expression of *ftz* requires both motifs, the DPE was found to regulate transcriptional onset, and the TATA-box to affect overall intensity. Our findings demonstrate that in the *tin* promoter, which lacks a natural TATA box, mutation in the DPE motif is sufficient to reduce overall nascent RNA levels (Fig. 8A). A comprehensive analysis of the regulation of transcriptional dynamics by endogenous promoter motifs will help to fully elucidate their fascinating roles during embryonic development.

The molecular mechanisms controlling heart formation are highly conserved in evolution from flies to humans (reviewed by Bodmer and Venkatesh, 1998; Cripps and Olson, 2002; Rotstein and Paululat, 2016). The vertebrate *tinman* homologue, *NKX2-5*, is required for heart specification and is expressed in early cardial progenitors (Harvey, 1996). Although vertebrate *NKX2-5* mutants are able to properly specify cardiac progenitor cells, the final organization of the heart is disturbed (Lyons et al., 1995; Targoff et al., 2013, 2008). Interestingly, additional *NKX2*-family members cannot compensate for the specific loss of *NKX2-5*, demonstrating the strong specific requirement of NKX2-5 and possibly its co-factors (Stutt et al., 2022).

The DPE motif was originally discovered as conserved from flies to human (Burke and Kadonaga, 1997), yet for many years, only a few human genes were experimentally shown to contain a functional DPE (Burke and Kadonaga, 1997; Duttke, 2014; Zhou and Chiang, 2001). Recently, machine learning models were used to define the downstream core promoter region (DPR) in human and *Drosophila* (Vo Ngoc et al., 2020, 2023). In parallel, preferred downstream positions required for proper transcriptional output were identified (PDP; Dreos et al., 2021). Interestingly, the core promoter of the human *NKX2-5* contains both the DPR and PDP motifs. It remains to be determined whether *NKX2-5* levels are controlled by its core promoter composition. If so, this would suggest that the regulatory function of the DPE during heart formation is not limited to *Drosophila* but is instead conserved, along with many components of the gene regulatory network. Notably, multiple cardiac pathologies (e.g. septal openings and conduction defects) result from mutations in the coding region of *NKX2-5* (Benson et al., 1999; Elliott et al., 2003; McElhinney et al., 2003; Schott et al., 1998). Thus, it is conceivable that, in addition to mutations in the protein coding region of *NKX2-5*, homozygous or heterozygous mutations in downstream core promoter motifs of *NKX2-5* could likewise be responsible for congenital heart defects.

In summary, we demonstrate the *in vivo* contribution of a single core promoter element, i.e. the DPE motif, to the regulation of the *tin* gene and its developmental gene regulatory network. This exemplifies the contribution of the endogenous core promoter to transcriptional regulation during *Drosophila melanogaster* embryogenesis and functional heart formation, thus paving the way for further exciting discoveries related to transcriptional regulation of developmental genes via their core promoter.

## MATERIALS AND METHODS

### Fly culture and stocks

Flies were cultured and crossed on standard media (cornmeal, yeast, molasses and agar) at 25°C, 60% relative humidity and under a 12 h light/12 h dark cycle. All the described embryonic development was performed at 25°C. F3 and M6 *tin^mDPE^* strains were generated based on a *white* co-conversion approach (Ge et al., 2016) using ssODN, as detailed by Levi et al. (2020). Cas9 is shorthand for the injected strain that was used as *tin^WT^* control in all the experiments. w; *tin^346^*/[TM3, *eve*-lacZ] is a balanced null allele described by Azpiazu and Frasch (1993).

### RNA extraction and real-time PCR analysis

Embryos (0-8 h) were collected and aged at 25°C as indicated. For each time interval, *tin^WT^* and two independent *tin^mDPE^* (F3, M6) strains were collected and processed in parallel. Total RNA was extracted from dechorionated embryos using the TRI Reagent (Sigma-Merck) according to the manufacturer's protocol, followed by ethanol precipitation for further purification. 1 μg RNA was further used for cDNA synthesis (qScript cDNA Synthesis Kit, Quantabio). Quantitative PCR using SYBR green (qPCRBIO SyGreen Blue Mix, PCR Biosystems) was performed using a StepOnePlus Real-Time PCR machine. Control reactions lacking reverse transcriptase were also performed to ensure that the levels of contaminating genomic DNA were negligible. Transcript levels were analyzed by the ΔΔCT method using Polr2F (RpII18) as an internal control. Each sample was run in triplicates. Statistical analysis was performed using ‘HH’ R package (https://CRAN.R-project.org/package=HH), with mean and standard deviation values exported from StepOnePlus software. Primer sequences are provided in Table S3.

### Western blot analysis

Protein extracts from 2-4 h, 4-6 h, 6-8 h, 8-10 h and 10-12 h dechorionated embryos were prepared in 2×DTT-based sample buffer at a final concentration of ∼0.5 mg embryos/µl. 15-20 µl of the sample was analyzed using 10% SDS-PAGE gel, followed by rabbit anti-Tinman polyclonal antibodies (1:1000 in 3% BSA; Yin et al., 1997) and then by goat-anti-rabbit IgG-HRP (1:5000 in 5% milk, Jackson ImmunoResearch). HRP signal was detected using an EZ-ECL kit (Biological Industries) or Luminata Crescendo Western HRP substrate (Mercury), and imaged using iBright Imaging System (Thermo). The use of the Tinman antibody results in background bands; however, the major band is above the 45 kDa size marker, as predicted. The same membrane was stripped (ST010, Gene Bio-Application) and re-blotted with mouse anti-Actin monoclonal antibodies (1:1000 in 3% BSA, Abcam ab8227) to ensure proper gel loading. Images were quantified using the iBright Analysis Software (Thermo); each sample was normalized to the detected Actin levels. Statistics was calculated with unpaired two-tailed one-sample *t*-test followed by Bonferroni correction for multiple testing.

### Immunostaining and staging *Drosophila* embryos

Dechorionated embryos were fixed in freshly prepared 1:1 mixture of heptane and 3.7% paraformaldehyde solution (diluted 1:10 in PBS) for 20 min with vigorous shaking. Devitellinization was performed in heptane:methanol 1:1 solution, and embryos were stored in methanol at −20°C. Before staining, embryos were washed three times in PBST (0.1% Tween-20 in PBS) and blocked in 2% BSA supplemented with 0.2% fetal calf serum. Embryos were incubated overnight at 4°C with the following primary antibodies: rabbit anti-Tinman (1:750; Yin et al., 1997), rabbit anti-Eve (1:800; Knirr and Frasch, 2001), rabbit anti-Mef2 (1:800; Bour et al., 1995), rabbit anti-Doc2 (1:2000, a generous gift from Dr Ingolf Reim, Philipps-University Marburg, Germany), rabbit anti-β3Tubulin (1:1500, a generous gift from Prof. Susanne Önel, Philipps-University Marburg, Germany), guinea pig anti-Odd (1:200, 805 from Asian Distribution Center for Segmentation Antibodies, distributed by Prof. Zeev Paroush, Hebrew University, Jerusalem, Israel), rat anti-Org1 (1:100, a generous gift from Dr Christoph Schaub and Dr Katrin Domsch, Heidelberg University, Germany), mouse anti-Svp (1:400, DSHB 5B11), mouse anti-β-galactosidase (1:1000, Promega, z3781) and chicken anti-β-galactosidase (1:500, Abcam, ab9361). Detection was performed using mainly goat anti-rabbit IgG H&L (DyLight 488) (1:500, Abcam ab96883), Cy5-goat anti-rabbit IgG H&L (1:1000, Abcam ab150075), Cy3-goat anti-mouse IgG H+L (1:500, Jackson ImmunoResearch 115-165-166), Cy3-donkey anti-guinea pig IgG H+L (1:1000, Jackson ImmunoResearch 706-165-148), goat anti-rat IgG H+L Alexa Fluor 488 (1:500, Thermo A-11006) and Cy3-goat anti-chicken IgY H+L (1:500, Abcam ab97145). Embryos were counterstained with Hoechst 33342 (Sigma-Aldrich), and mounted in n-propyl gallate-based anti-fade mounting medium [5% w/v n-propyl gallate dissolved in 0.1 M Tris (pH 9) and glycerol in a 1:9 ratio]. Images were acquired with a Leica SP8 confocal microscope, using oil immersion objectives. *Z*-stack maximal projections are shown. Cardioblast cells were counted using the ImageJ software, followed by statistical analysis conducted using R. For *tin^WT^* or *tin^mDPE^* crosses with *tin^346^*/TM3, the embryos resulting from the same cross were used. β-Gal staining was used to distinguish between wild type (TM3) and *tin^346^* allele.

Bownes developmental stages were used for embryo development classification (after José and Campos-Ortega, 1985 and www.sdbonline.org/sites/fly/aimain/2stages.htm). *tinman in situ* images were obtained from Berkeley *Drosophila* Genome Project (Tomancak et al., 2002, 2007) via the FlyExpress website (Kumar et al., 2011).

### Viability testing

*tin^WT^* and *tin^mDPE^* (F3 and M6) virgins were crossed to *tin^346^*/[TM3 (Sb), *eve*-lacZ] in triplicates (biological replicates), and each vial was flipped three times (technical replicates). Parental flies were discarded; F1 flies were anesthetized, separated based on Sb phenotype, counted in groups of 5 and then discarded. Each vial was counted twice, ensuring most of the eclosed flies are scored. For analysis, non-Sb to Sb ratios were log2-transformed. One-way nested ANOVA was performed to test the effect of strain on non-Sb/Sb ratios. Specifically, a linear mixed effect model was performed, and the ANOVA was performed on the resulting model. Post-hoc analysis was performed as pairwise comparisons using Tukey's method.

### Adult *Drosophila* heart assay

All dissection steps were carried out using artificial hemolymph. In brief, 3-week-old female flies were anesthetized with FlyNap (Carolina Biological), transferred to a petroleum jelly-coated Petri dish, and dissected as described previously (Vogler and Ocorr, 2009). The dissected hearts were equilibrated for 15 min at room temperature under constant oxygenation. High-speed movies were captured on an Olympus BX61WI microscope with a 10× immersion objective, using a Hamamatsu Orca Flash4 CMOS digital camera and HCI image capture software (Hamamatsu). Movies were then analyzed with custom-designed software (Ocorr et al., 2009) to determine physiological heart parameters, including diameters.

### Immunostaining of adult *Drosophila* hearts

Adult hearts were dissected, fixed and stained according to Alayari et al. (2009). To label cardiac tissue to determine heart size and structure, mouse anti-α-Spectrin (DSHB 3A9, 1:40) and Alexa Fluor 633 phalloidin (1:1000, Thermo Fisher) were used. Samples were imaged on a Zeiss Imager Z1 and Apotome 2. Image stacks were analyzed using FIJI/ImageJ (Schindelin et al., 2012). For heart diameters, the distance between heart walls was measured in segment A2 posterior to the ostia cells. All statistical analysis and graph plotting was carried out using R.

### FlyBowl experiments

In this study, *tin^WT^* flies were used as the wild-type strain, while *tin^mDPE^* (F3 and M6) lines served as *tin^mDPE^* strains. Flies were raised at 25°C with 60-70% relative humidity under a 12-h light/dark cycle, and maintained on a standard diet of cornmeal, yeast, molasses and agar. Virgin flies were lightly anesthetized with CO_2_ and collected shortly after hatching. Groups of ten flies were housed under the same conditions as their parents until the final experimental stage, which occurred at three time points: after 4, 9 and 21 days. Behavioral experiments were conducted within 2 h of the onset of light. Each group of ten flies was placed in a FlyBowl arena (Bentzur et al., 2021; Kabra et al., 2013), and their activity was recorded over a 15-min period using the FlyBowl Data Capture (FBDC) software. Fly orientation, position and trajectories were tracked using CTRAX, and tracking errors were corrected with a custom MATLAB software called FixTRAX. Activity data were classified using the machine learning tool JAABA. Data normality was assessed with the Shapiro-Wilk test, and normally distributed data were analyzed using one-way ANOVA followed by Tukey's range test to determine significant differences between experimental conditions.

### csRNA-seq samples and processing

*tin^WT^* and *tin^mDPE^* (F3 and M6) embryos were aged at 25°C and collected at 0-2 h, 2-4 h, 4-6 h and 6-8 h time intervals. Total RNA was extracted from dechorionated embryos using the TRI Reagent (Sigma-Merck) according to the manufacturer's protocol, followed by ethanol precipitation for further purification. Reduction of *tin* levels was verified using RT-qPCR, and samples were subjected to csRNA-seq analysis protocol version 5.2 (Duttke et al., 2022). Briefly, RNA was heat denatured and short RNAs (18-65 nucleotides) purified by 15% UREA-PAGE. A small fraction (5%) of these short RNAs was used to generate input libraries (conventional small RNA-seq), and the remainder was cap selected with 5′ monophosphate-dependent exonuclease (TER51020) followed by two phosphatase (CIP) treatments. Sequencing libraries for sRNA-seq and csRNA-seq were generated using the NEB sRNA kit, but with addition of RppH for decapping (Hetzel et al., 2016).

Sequencing data were analyzed using HOMER csRNAseq module (http://homer.ucsd.edu/homer/ngs/csRNAseq/index.html (Duttke et al., 2019) and R custom scripts. 3′ adapter sequences of the reads were trimmed using HOMER (Heinz et al., 2010) and aligned to *dm6* genome using STAR (version 2.7.10a) (Dobin et al., 2013). Reads were visualized as strand-specific bedGraph using HOMER makeUCSCfile command with -style tss parameter. Peak calling was performed using the findcsRNATSS.pl function in HOMER (Duttke et al., 2019), with input RNA-seq used as background to eliminate transcripts from degraded and high-abundance RNAs in csRNAseq. HOMER annotatePeaks.pl command was used with -rlog parameter for calculating the normalized expression values for each peak used in downstream analyses. It was also used for generating transcription profile plots, e.g. annotatePeaks.pl tss dm6 -size 400 -hist 10 -pc 3. For differential expression, getDiffExpression.pl -edgeR -simpleNorm -dispersion 0.05 -AvsA was used on raw counts. ComplexHeatmap R package (Gu et al., 2016) was used for hierarchical clustering. HOMER analyzeClusters.pl was used for motifs and GO terms enrichment analysis in the identified cluster. plotPCA function from DESeq2 package was used with parameter *ntop=40,000.* Raw sequence data were deposited in the NCBI GEO database under accession number GSE221852.

### Motif enrichment analysis

For each time interval, the list of peaks with pAdj<0.1 for both *tin^mDPE^* (F3 and M6) versus *tin^WT^* was extracted based on differential expression analysis (above). The ‘combined’ list comprises the unique list of differentially expressed peaks within at least one time interval. Peak coordinates were used for construction of BED files, and sequences were extracted based on dm6 genome. MEME analysis (Bailey and Elkan, 1994) was performed on each list separately using the following command: meme -dna -maxsize 5000000 [listName] -o [listName_outDir] -minw 5 -nmotifs 10. The DNA sequences that were used as input for the MEME analysis are included in Table S4. For the analysis, only peaks with ‘promoter’ annotation were used; however, similar results were obtained when using all the differentially expressed peaks. Over-represented motifs were converted to HOMER format, which was then used to scan the relevant BED files with the motifs of interest.

## Supplementary Material



10.1242/develop.202355_sup1 Supplementary information

Table S1. Quantification of Tinman protein levels using western blot analyses. Protein extracts were prepared from embryos collected at 2-4h, 4–6h, 6-8h, 8-10h or 10-12h time intervals and subjected to western blot analyses. For each membrane, embryos from the same fly populations were collected. Western blotting of each membrane was initially performed using rabbit anti-Tinman antibodies. The levels of Actin as a loading control were detected using mouse anti-Actin antibodies. n=5-6 biological replicates.

Table S2. Summary of expression profiles and selected GO terms for each cluster, used for Fig. 8.

Table S3. RT-qPCR primers used in the manuscript.

Table S4. Sequences of differentially expressed csRNA-seq peaks used as input for MEME utility
