# Supplementary material for: From promoter motif to cardiac function: a single DPE motif affects transcription regulation and organ function in vivo
Source: Development. 2024 Jul 29;151(14):dev202355. doi: 10.1242/dev.202355 (PMC11317100; doi:10.1242/dev.202355)
Supplement: Supplementary information [file develop-151-202355-s1.pdf]

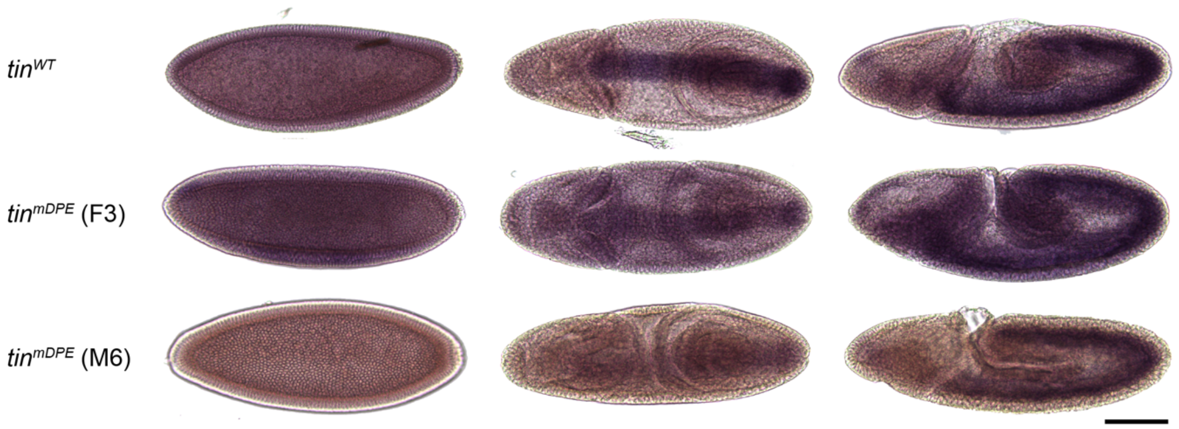

Fig. S1. *tinman* expression pattern is similar in early (<stage 8) *tin*^{WT} and *tin*^{mDPE} embryos. *In situ* RNA hybridization using a DIG-labeled *tinman* probe resulted in no detectable difference of the staining pattern for 0-4h embryos (Bownes stage <8).

Embryos were dechorionated in bleach and fixed in 4% formaldehyde/PBS/heptane for 20 minutes. pFlc-1-tin plasmid RE01329 (DGRC Stock 7896; https://dgrc.bio.indiana.edu//stock/7896; RRID:DGRC_7896) was linearized with NotI and transcribed in the presence of DIG labeling mix (Roche) to create a DIG-UTP labeled antisense RNA probe. Expression of *tin* was visualized by whole-mount *in situ* hybridization using the DIG-labeled probe detected by anti-DIG antibodies conjugated to alkaline phosphatase (Roche). Whole-mount *in situ* hybridization procedure was carried out essentially as described in (Wilk et al., 2010). Specimens were mounted in 70% glycerol/PBS and imaged within a week. DIC microscope images were acquired with a Leica LMD7 microscope using a 10X objective lens. All embryos were imaged under the same conditions. Scale bar = 100µm.

Development • Supplementary information

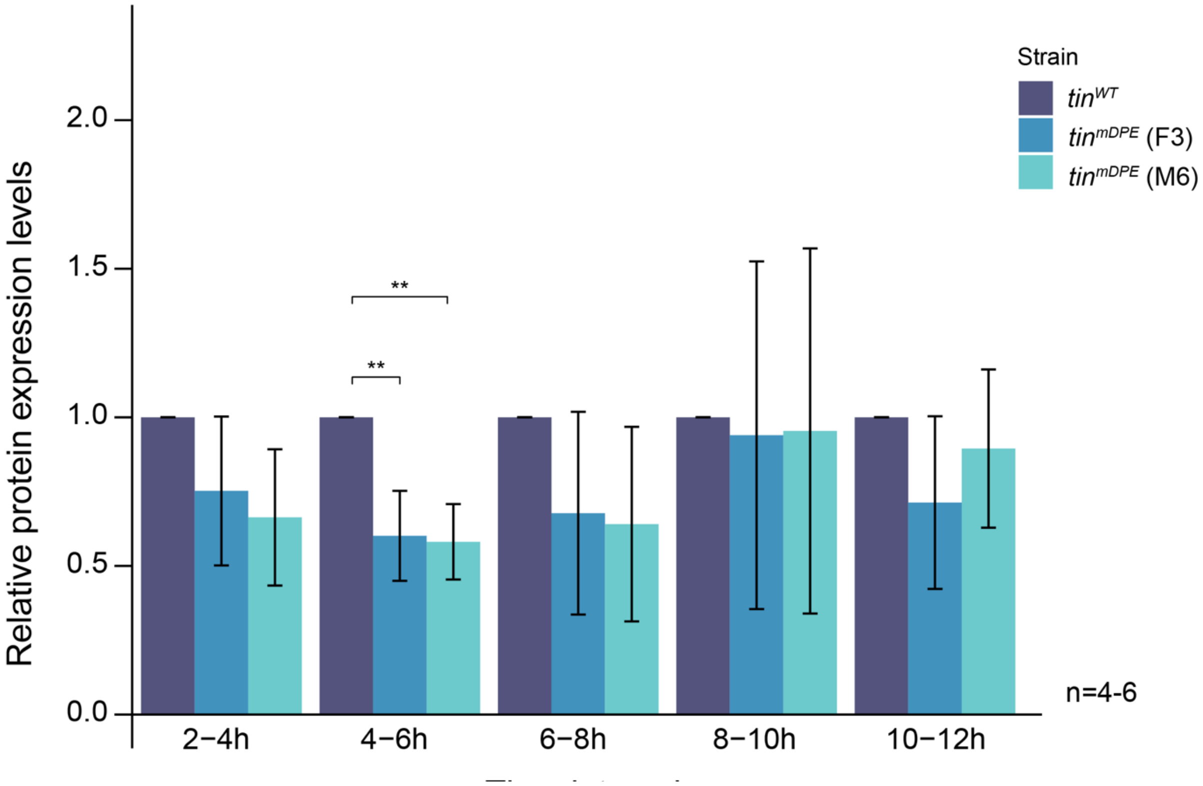

Fig. S2. Tinman protein levels were significantly lower in 4–6h *tin^{mDPE}* embryos compared to *tin^{WT}*. Quantification of Tinman protein levels using western blot analyses. Protein extracts were prepared from embryos collected at 2-4h, 4–6h, 6-8h, 8-10h or 10-12h time intervals. For each membrane, embryos from the same fly populations were collected. Western blotting of each membrane was initially performed using rabbit anti-Tinman antibodies. The levels of Actin as a loading control were detected using mouse anti-Actin antibodies. n=4-6 biological replicates. Error bars represent the STD. $^{*}p \leqslant 0.5$, $^{**}p \leqslant 0.01$, $^{***}p < 0.001$, unpaired two-tailed one-sample t-tests followed by Bonferroni correction for multiple testing; Asterisks denote statistically significant changes of Tinman levels in *tin^{mDPE}* compared to *tin^{WT}*.

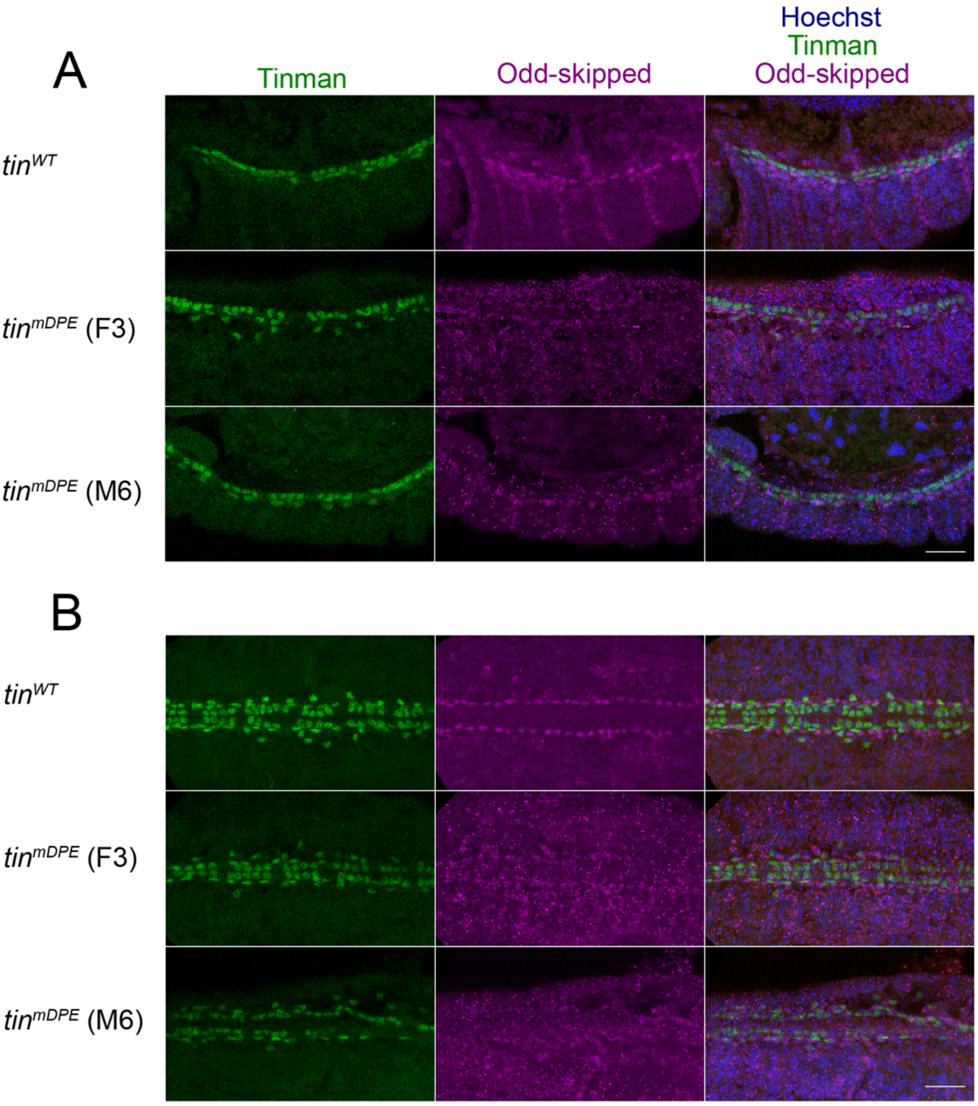

Fig. S3. Tinman and Odd staining of the mDPE embryos. Embryos from *tin*^{WT}, *tin*^{mDPE} (F3, M6) were stained for Tinman (green) and Odd-skipped (magenta). (A) Stage 13 and (B) Stage 17 embryos. Z-stack maximal projections are shown, scale bar = 25µm.

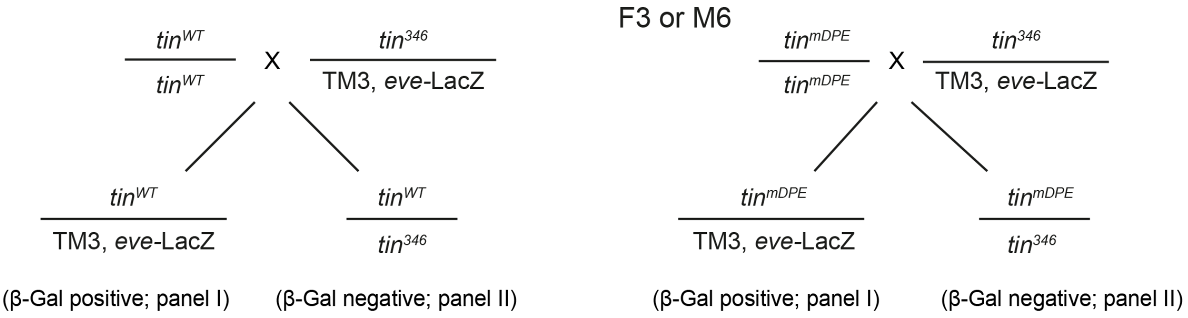

Fig. S4. Crossing scheme for each *tin*^{WT} and *tin*^{mDPE} (F3 and M6) homozygous strains to the *tin*^{346}/[TM3, *eve*-LacZ] strain. Embryos resulting from each cross were co-stained for β-Gal to identify β-Gal-positive embryos, *i.e.*, *tin*^{WT}/[TM3, *eve*-LacZ] and *tin*^{mDPE}/ [TM3, *eve*-LacZ] (panels I in Figs. 4,5 and Fig. S5) and β-Gal-negative embryos, *i.e.*, *tin*^{WT}/*tin*^{346} and *tin*^{mDPE}/*tin*^{346} (panels II in Figs. 4,5 and Fig. S5).

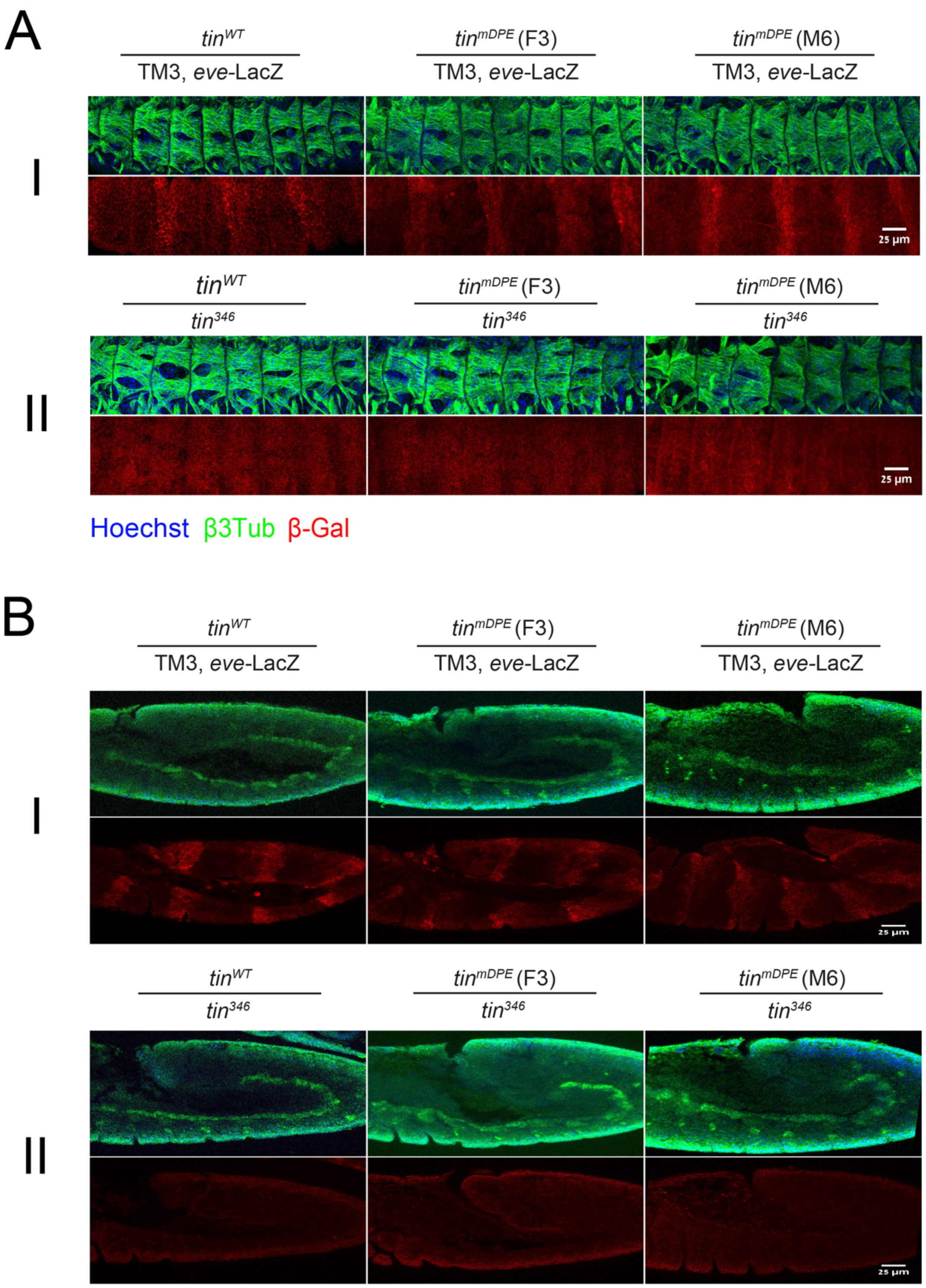

Fig. S5. The expression patterns of β3Tubulin, a marker of somatic musculature, and Org1, a marker for visceral musculature, are unaffected in *tin*^{mDPE} embryos when tested *in trans* to the *tin* null mutation. *tin*^{WT} and *tin*^{mDPE} (F3, M6) strains were crossed to the *tin*^{346}/ [TM3, *eve*-LacZ] strain. Embryos resulting from each cross were co-stained for β-Gal to identify β-Gal-positive embryos, *i.e.*, *tin*^{WT}/[TM3, *eve*-LacZ] and *tin*^{mDPE}/ [TM3, *eve*-LacZ]

Development • Supplementary information

(panels I) and β-Gal-negative embryos, *i.e.*, tin^{WT}/tin^{346} and tin^{mDPE}/tin^{346} (panels II). (A) Embryos from all three crosses were stained using anti-β3Tubulin (green), anti-β-Gal (red) and counterstained with a nuclear dye (Hoechst, blue). One representative embryo (stages ~14-16) is shown for each fly line. Z-stack 3D projections are shown. Anterior to the left. Scale bar = 25μm. (B) Embryos from all three crosses were stained using anti-Org1 (green), anti-β-Gal (red) and counterstained with a nuclear dye (Hoechst, blue). One representative embryo (stages ~11-13) is shown for each fly line. Z-stack 3D projections are shown. Anterior to the left. Scale bar = 25μm.

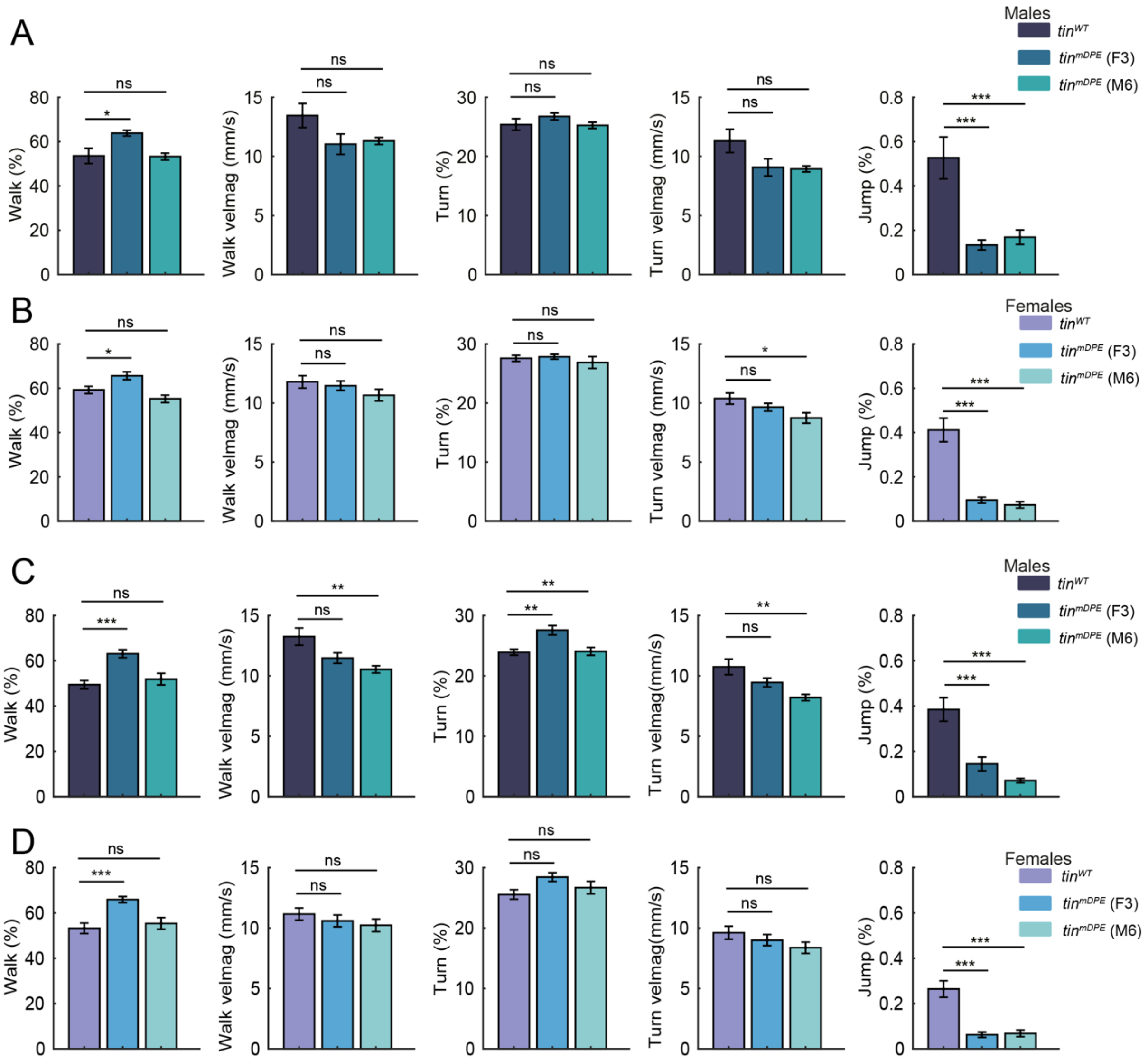

Fig. S6. Mutation of endogenous *tinman* DPE affects distinct activity features of 4- and 9-day-old flies. The locomotor activity of (A-B) 4 day-old and (C-D) 9-day old tin^{WT} line (purple) and tin^{mDPE} lines (F3 - blue and M6 - turquoise), was measured using the FlyBowl system. The average percentage of time flies spent walking, changing orientation (turning), jumping, and their average velocity during walking and turning are depicted for males (A,C) and females (B,D). n=9 (10 flies/arena). One-Way ANOVA followed by Tukey's $^{*}p < 0.05$, $^{**}p < 0.01$, $^{***}p < 0.001$. Error bars signify SEM. Only comparisons to the tin^{WT} samples are presented.

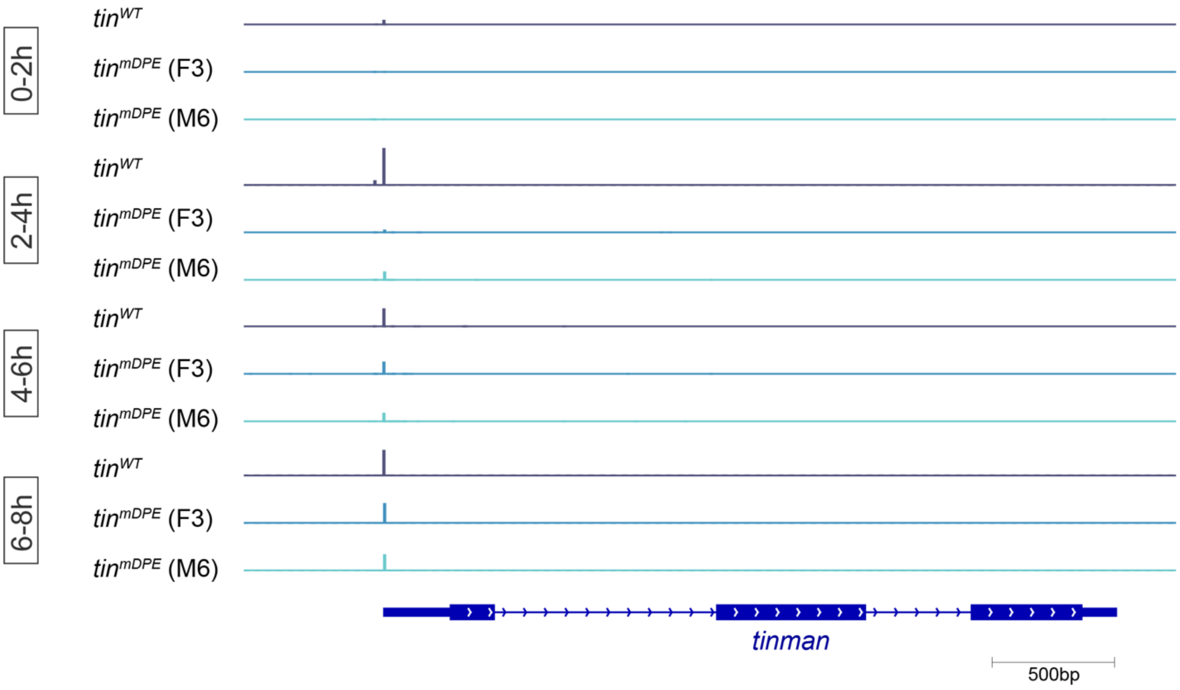

Fig. S7. The *tin* mDPE allele does not result in additional TSSs. Nascent transcription profile of the whole *tinman* locus. Depicted region is Chr3R:21378500-218200 (dm6), scale is 0 to 530 for all tracks.

Development • Supplementary information

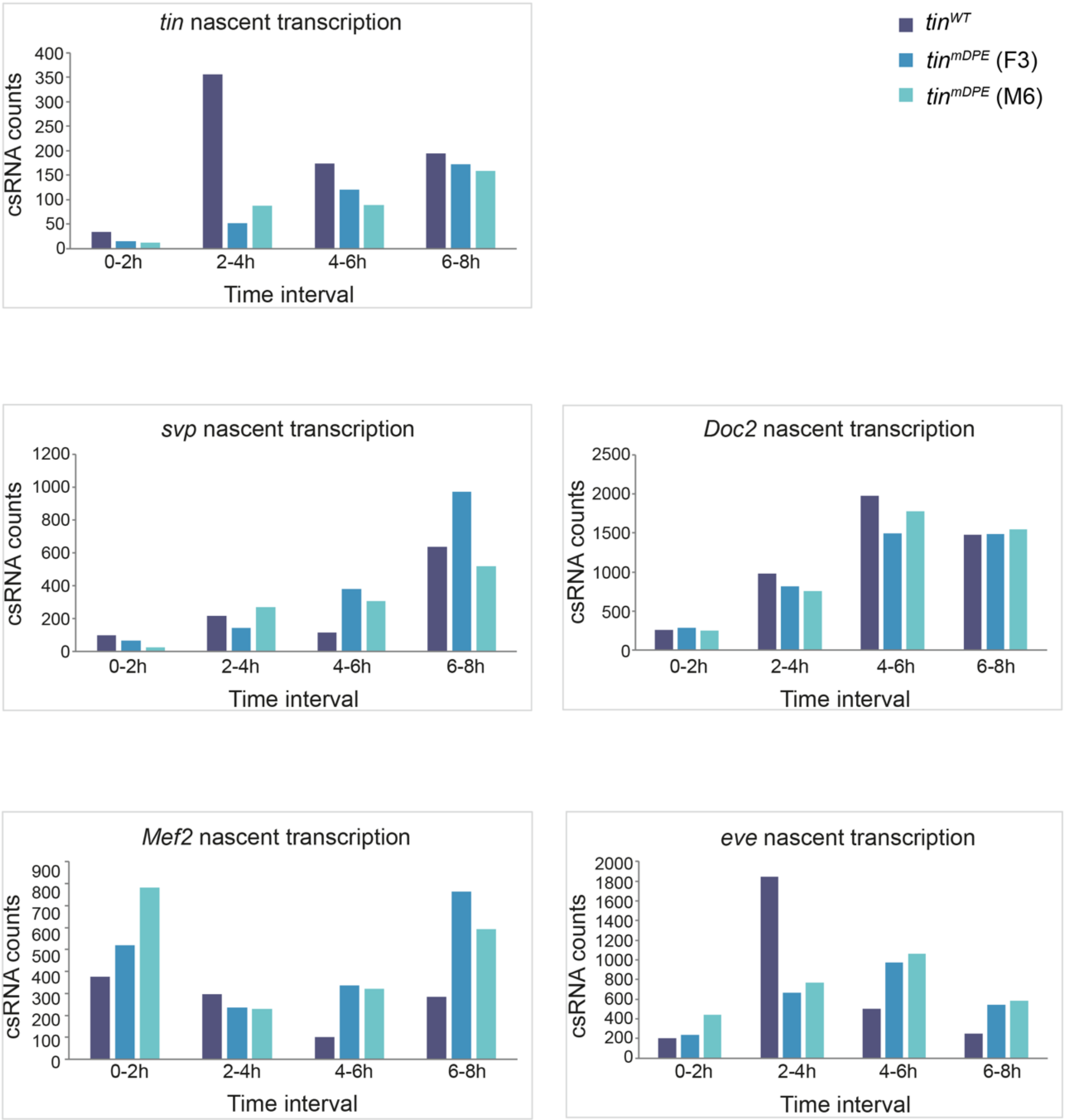

Fig. S8. Nascent RNA counts at 4-6h of *tin* and its target genes *Doc2, Mef2* and *eve* are similar to their expression levels at 4-6h analyzed by RT-qPCR. Graphs depict nascent RNA (csRNA) expression levels of *tin*, *svp*, *Doc2*, *Mef2* and *eve* at 0–2h, 2–4h, 4–6h and 6–8h time intervals of *tin*^{WT} and *tin*^{mDPE} (F3 and M6) embryos.

Development • Supplementary information

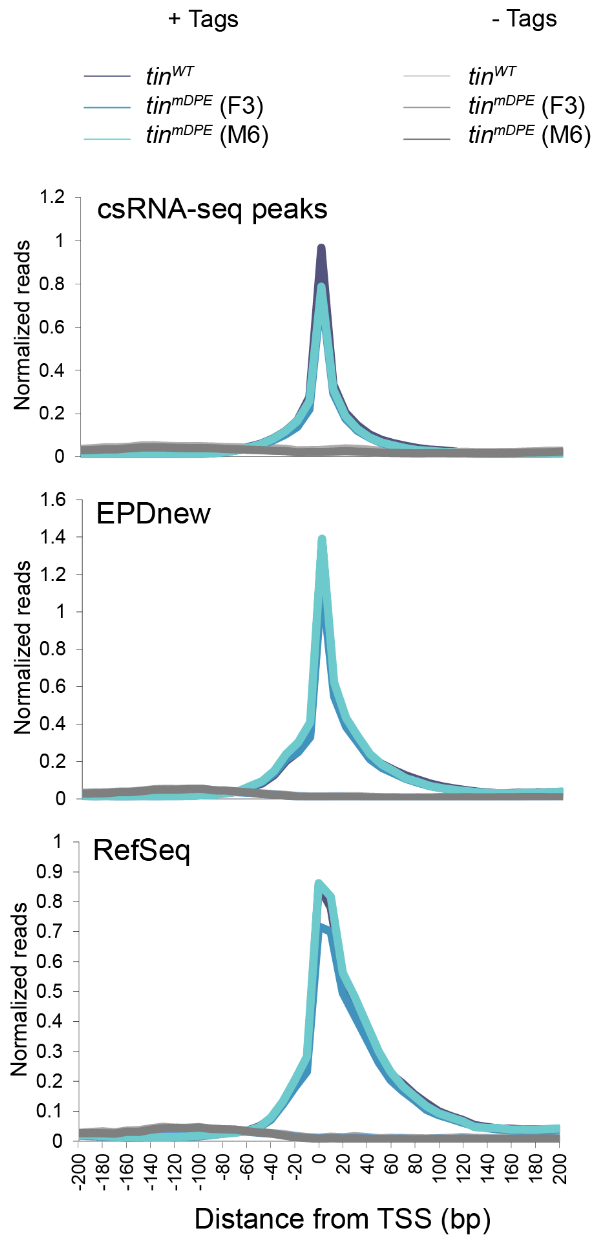

Fig. S9. The choice of TSSs source result in slightly different nascent transcription profiles. The same csRNA-seq data (2-4h embryos of *tin*^{WT} and *tin*^{mDPE} - F3 and M6 strains) was mapped using called csRNA-seq peaks, EPDnew or RefSeq. Note that csRNA-seq reads exhibited rather symmetrical distribution around EPDnew TSSs as compared to HOMER's default (RefSeq). Transcription initiation seems to be detectable at sequences downstream of RefSeq-defined TSSs. In contrast, EPDnew-based TSSs exhibit a more symmetric distribution, similar to the csRNAseq-based TSSs. Divergent transcription (- tags) is barely detectable, consistent with previous reports that divergent transcription is predominantly absent in *Drosophila melanogaster* (Core et al., 2012; Meers et al., 2018).

Development • Supplementary information

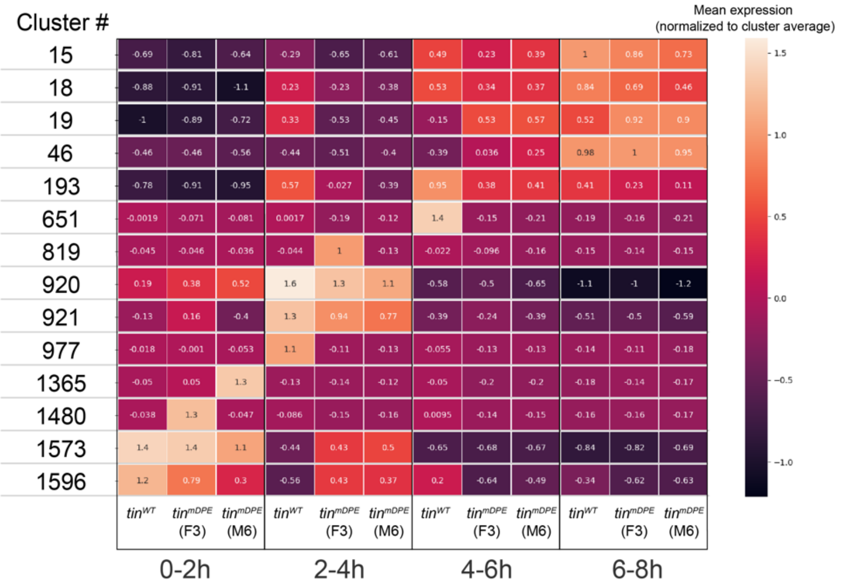

Cluster #	Count	BestGO	BestMotif
15	652	post-embryonic animal organ development|GO:0048569|1.97e-35	Unknown1(NR|MYGGTCACACTG|1e-11
18	575	post-embryonic animal organ development|GO:0048569|5.92e-31	Trl(Zf)|RGAGAGAG|1e-11
19	451	sensory organ development|GO:0007423|6.42e-23	M1BP(Zf)|CAGTGTGACCGT|1e-14
46	434	cell fate commitment|GO:0045165|6.24e-16	Trl(Zf)|RGAGAGAG|1e-4
193	322	cell fate commitment|GO:0045165|8.16e-12	DREF|AVYTATCGATAD|1e-5
651	408	dorsal appendage formation|GO:0046843|6.84e-04	Unknown2|CATCMCTA|1e-3
819	382	cell morphogenesis involved in differentiation|GO:0000904|6.21e-07	Zelda(Zf)|KBCTACCTGW|1e-9
920	327	stem cell fate commitment|GO:0048865|4.35e-06	Zelda(Zf)|KBCTACCTGW|1e-39
921	330	cell fate commitment|GO:0045165|1.96e-09	Zelda(Zf)|KBCTACCTGW|1e-22
977	846	regulation of cell differentiation|GO:0045595|6.31e-13	Zelda(Zf)|KBCTACCTGW|1e-5
1365	941	cell projection morphogenesis|GO:0048858|1.54e-08	TATA-box|CTATAAAAGCSV|1e-1
1480	1148	wing disc morphogenesis|GO:0007472|7.92e-07	dHNF4(NR)|GGTCCAAAGTCCAMT|1e-1
1573	1436	cytoplasmic translation|GO:0002181|1.27e-16	Unknown1(NR|MYGGTCACACTG|1e-13
1596	361	positive regulation of protein metabolic process|GO:0051247|5.30e-09	DREF|AVYTATCGATAD|1e-47

Fig. S10. Full output of the analyzeClusters.pl script. Each cluster is annotated with cluster number, number of consisting peaks (n), top enriched HOMER motif and GO term. For each sample, the mean average expression of peaks in the cluster was normalized by the average expression per cluster (derived from all the samples). The following command was used: *analyzeClusters.pl -i all.rlog.byTime.txt -o ./cluster.allRlog.c300.t8/ -peaks -minDiff 1 -center -genome dm6 -size -100,100 -thresh -0.8 -min 300 -cpu 30.*

Development • Supplementary information

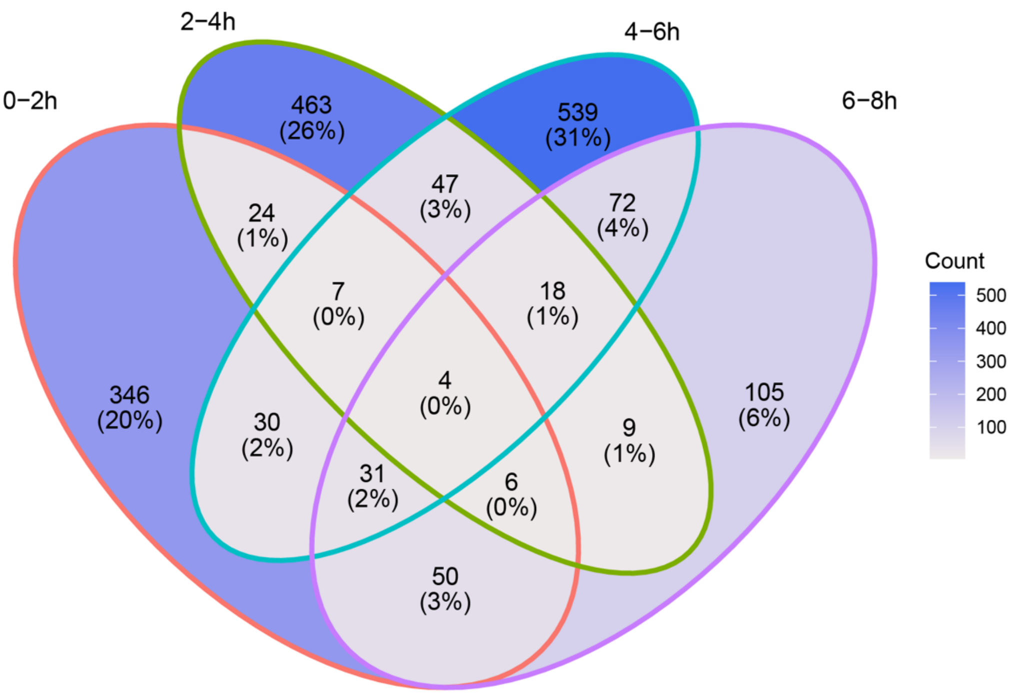

Fig. S11. The majority of differentially expressed csRNA-seq peaks do not overlap across the tested time points. The overlap between differentially expressed peaks for each time point was examined using ggVennDiagram R package (Gao et al., 2021). Each section is colored according to the number of peaks.

Development • Supplementary information

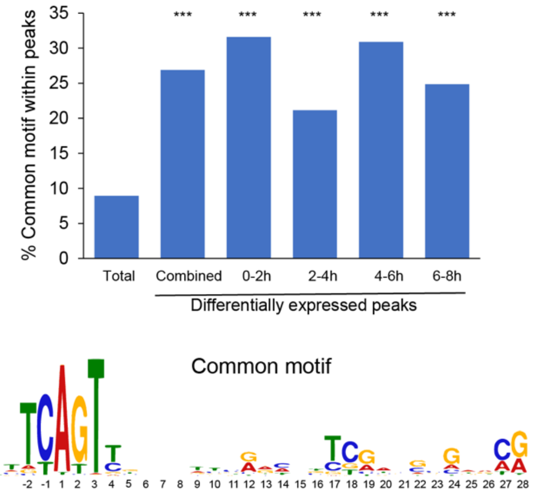

Fig. S12. The DPE motif is enriched in differentially expressed genes following *tinman* DPE mutation. The common motif enriched among all the differentially expressed promoters (bottom) is enriched above the expected background (total peaks) in all differentially expressed peaks (combined), as well as in each individual time interval (0-2h, 2-4h, 4-6h, 6-8h). ***p< 0.001, one proportion Z-test.

Development • Supplementary information

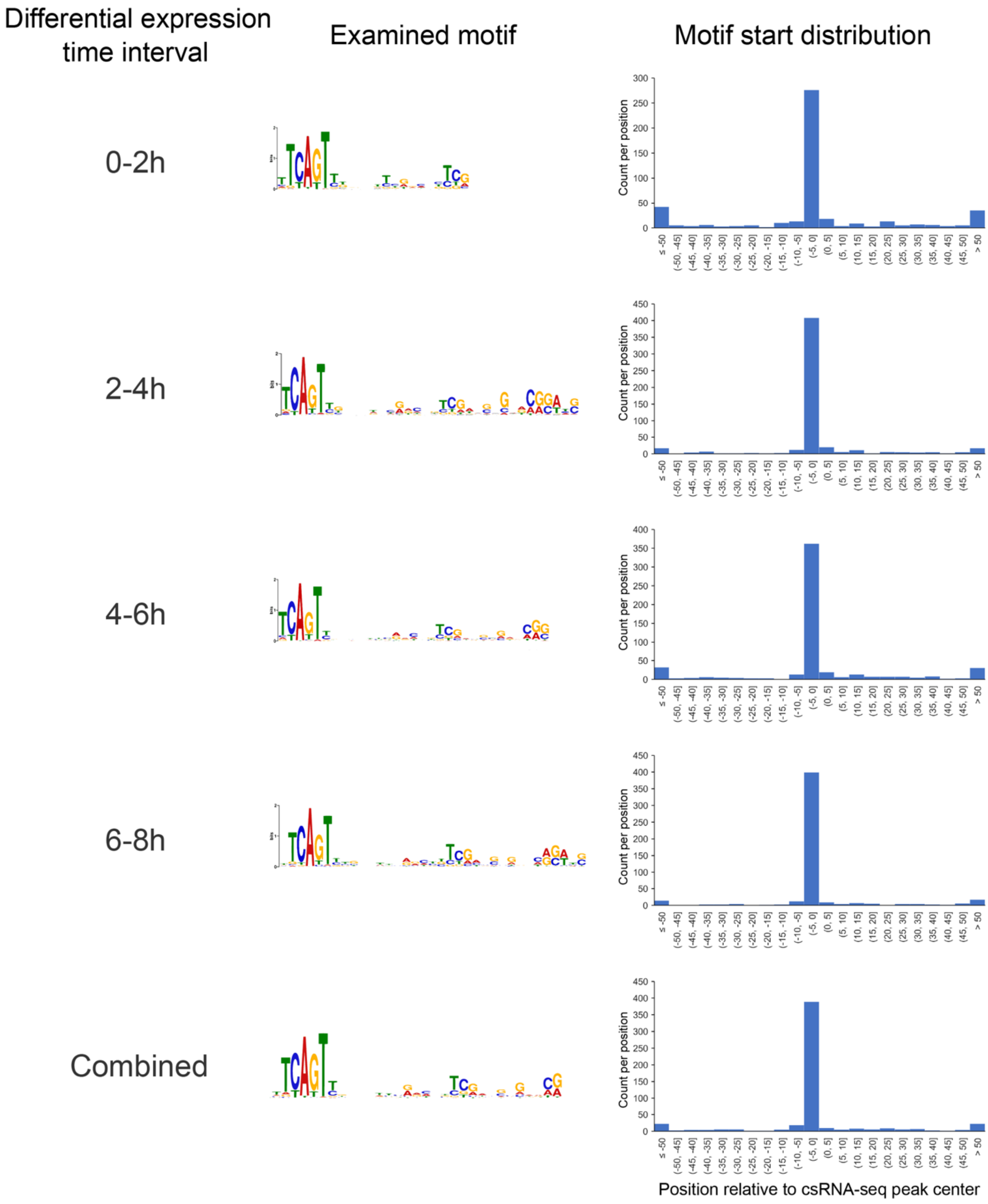

Fig. S13. The detected Inr+DPE motifs are strictly positioned relative to the differentially expressed csRNA-seq peaks. The lists of differentially expressed peaks at each time point, and at all time points combined, were scanned using the Inr+DPE-like motifs detected for each time point (shown on the right, same as in Fig. 8A). A specific enrichment at positions ±5bp is evident for all motifs. The indicated positions (x-axis) are of the motif start relative to the detected csRNA-seq peak center.

Development • Supplementary information

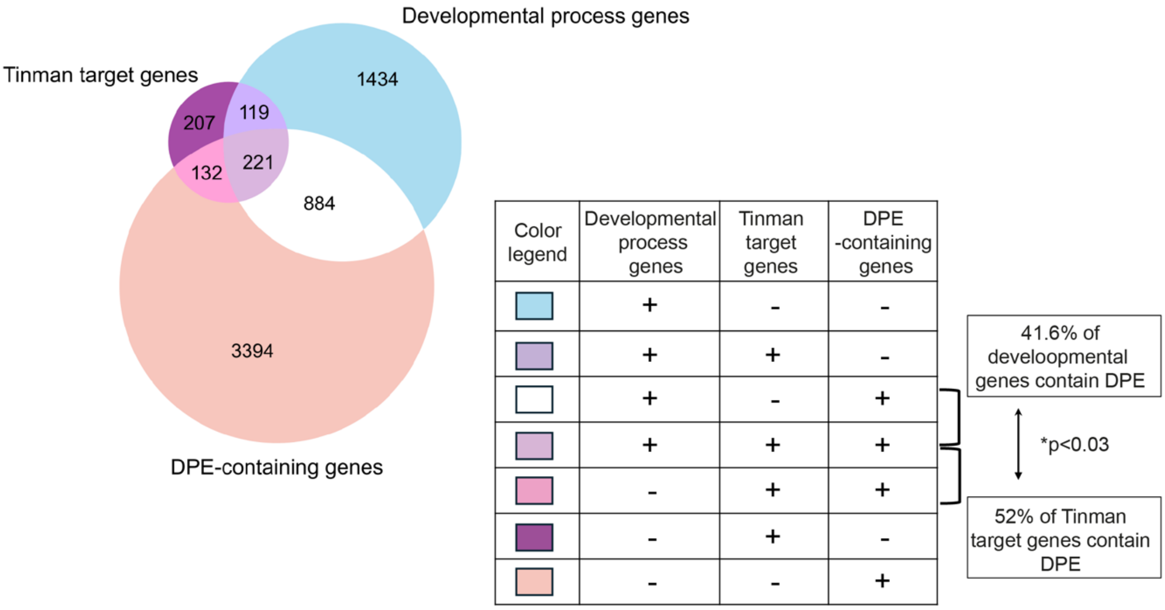

Fig. S14. Tinman target genes are significantly enriched for DPE motifs. The weighted venn diagram depicts the number of genes overlapping between 3 gene subsets: Tinman target genes, *Drosophila melanogaster* genes that are classified under the developmental process GO term, and DPE containing genes. Tinman target genes previously identified by Chromatin Immunoprecipitation (ChIP) (Jin et al., 2013) were retrieved from GEO database GSE41628. DPE-containing genes were identified by ElemeNT 2023 (Adato et al., 2024). Developmental process GO term included genes retrieved from Panther (Annotation Version and Release Date: GO Ontology database DOI: 10.5281/zenodo.10536401 Released 2024-01-17). All gene names were converted to FlyBase names. Statistical significance was analyzed by the chi-square test.

Development • Supplementary information

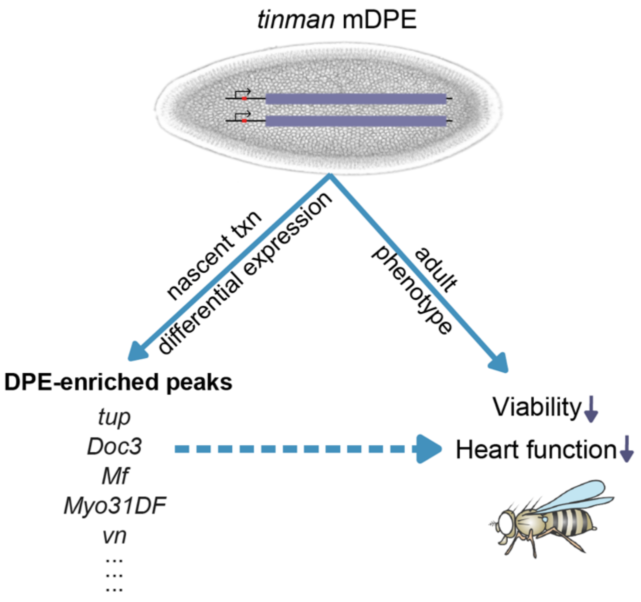

Fig. S15. Schematic summary. Endogenous *tinman* promoter was genetically edited using CRISPR to change the 7bp encompassing the DPE motif of the *tinman* promoter. This genomic editing resulted in viable homozygotes, which present major changes in nascent transcription (txn) of muscle and developmental genes, enriched for DPE-like motifs in their core promoter. In addition, the *tinman* mDPE homozygotes present impaired viability and heart function parameters, demonstrating the *in vivo* importance of a single core promoter element, the DPE motif. Schematic fly image is from Roote and Prokop (2017) (see also Roote and Prokop, 2013).

Development • Supplementary information

Table S1. Quantification of Tinman protein levels using western blot analyses. Protein extracts were prepared from embryos collected at 2-4h, 4–6h, 6-8h, 8-10h or 10-12h time intervals and subjected to western blot analyses. For each membrane, embryos from the same fly populations were collected. Western blotting of each membrane was initially performed using rabbit anti-Tinman antibodies. The levels of Actin as a loading control were detected using mouse anti-Actin antibodies. n=5-6 biological replicates.

Available for download at
https://journals.biologists.com/dev/article-lookup/doi/10.1242/dev.202355#supplementary-data

Table S2. Summary of expression profiles and selected GO terms for each cluster, used for Fig. 8.

Available for download at
https://journals.biologists.com/dev/article-lookup/doi/10.1242/dev.202355#supplementary-data

Table S3. RT-qPCR primers used in the manuscript.

Available for download at
https://journals.biologists.com/dev/article-lookup/doi/10.1242/dev.202355#supplementary-data

Table S4. Sequences of differentially expressed csRNA-seq peaks used as input for MEME utility

Available for download at
https://journals.biologists.com/dev/article-lookup/doi/10.1242/dev.202355#supplementary-data

REFERENCES

Adato, O., Sloutskin, A., Komemi, H., Brabb, I., Duttke, S., Bucher, P., Unger, R. and Juven-Gershon, T. (2024). ElemeNT 2023: an enhanced tool for detection and curation of core promoter elements. *Bioinformatics* **40**.

Core, L. J., Waterfall, J. J., Gilchrist, D. A., Fargo, D. C., Kwak, H., Adelman, K. and Lis, J. T. (2012). Defining the status of RNA polymerase at promoters. *Cell Rep* **2**, 1025-1035.

Gao, C. H., Yu, G. and Cai, P. (2021). ggVennDiagram: An Intuitive, Easy-to-Use, and Highly Customizable R Package to Generate Venn Diagram. *Front Genet* **12**, 706907.

Jin, H., Stojnic, R., Adryan, B., Ozdemir, A., Stathopoulos, A. and Frasch, M. (2013). Genome-wide screens for in vivo Tinman binding sites identify cardiac enhancers with diverse functional architectures. *PLoS genetics* **9**, e1003195.

Meers, M. P., Adelman, K., Duronio, R. J., Strahl, B. D., McKay, D. J. and Matera, A. G. (2018). Transcription start site profiling uncovers divergent transcription and enhancer-associated RNAs in Drosophila melanogaster. *BMC Genomics* **19**, 157.

Roote, J. and Prokop, A. (2013). How to design a genetic mating scheme: a basic training package for Drosophila genetics. *G3* **3**, 353-358.

Wilk, R., Murthy, S. U. M., Yan, H. and Krause, H. M. (2010). In Situ Hybridization: Fruit Fly Embryos and Tissues. *Current Protocols Essential Laboratory Techniques* **4**, 9.3.1-9.3.24.

Development • Supplementary information
