# Supplementary material for: From promoter motif to cardiac function: a single DPE motif affects transcription regulation and organ function in vivo
Source: Development. 2024 Jul 29;151(14):dev202355. doi: 10.1242/dev.202355 (PMC11317100; doi:10.1242/dev.202355)
Supplement: Supplementary file 1 [file develop-151-202355_review_history.pdf]

From promoter motif to cardiac function: a single DPE motif affects transcription regulation and organ function *in vivo*

Anna Sloutskin, Dekel Itzhak, Georg Vogler, Hadar Pozeilov, Diana Ideses, Hadar Alter, Orit Adato, Hadar Shachar, Tirza Doniger, Galit Shohat-Ophir, Manfred Frasch, Rolf Bodmer, Sascha H Duttke and Tamar Juven-Gershon
DOI: 10.1242/dev.202355

Editor: Benoit Bruneau

Review timeline
Original submission: 18 September 2023
Editorial decision: 1 December 2023
First revision received: 5 June 2024
Accepted: 26 June 2024

Original submission

First decision letter

MS ID#: DEVELOP/2023/202355

MS TITLE: From promoter motif to cardiac function: A single DPE motif affects transcription regulation and organ function in vivo

AUTHORS: Anna Sloutskin, Dekel Itzhak, Georg Vogler, Diana Ideses, Hadar Alter, Hadar Shachar, Tirza Doniger, Manfred Frasch, Rolf Bodmer, Sascha H Duttke, and Tamar Juven-Gershon

I have now received all the referees' reports on the above manuscript, and have reached a decision. The referees' comments are appended below, or you can access them online: please go to BenchPress and click on the 'Manuscripts with Decisions' queue in the Author Area.

As you will see, the referees express considerable interest in your work, but have some significant criticisms and recommend a substantial revision of your manuscript before we can consider publication. If you are able to revise the manuscript along the lines suggested, which may involve further experiments, I will be happy receive a revised version of the manuscript. Your revised paper will be re-reviewed by one or more of the original referees, and acceptance of your manuscript will depend on your addressing satisfactorily the reviewers' major concerns. Please also note that Development will normally permit only one round of major revision. If it would be helpful, you are welcome to contact us to discuss your revision in greater detail. Please send us a point-by-point response indicating your plans for addressing the referees' comments, and we will look over this and provide further guidance.

Please attend to all of the reviewers' comments and ensure that you clearly highlight all changes made in the revised manuscript. Please avoid using 'Tracked changes' in Word files as these are lost in PDF conversion. I should be grateful if you would also provide a point-by-point response detailing how you have dealt with the points raised by the reviewers in the 'Response to Reviewers' box. If you do not agree with any of their criticisms or suggestions please explain clearly why this is so.

Reviewer 1

© 2024. Published by The Company of Biologists under the terms of the Creative Commons Attribution License (https://creativecommons.org/licenses/by/4.0/).

Advance summary and potential significance to field

Journal: Development MS ID#: DEVELOP/2023/202355 From promoter motif to cardiac function: A single DPE motif affects transcription regulation and organ function in vivo

The manuscript by Sloutskin et al. uses Drosophila melanogaster to study the effects of mutating the downstream core promoter element (DPE) of the endogenous tin gene. The authors used CRISPR to create two independent mutant strains and found reduced tin RNA and protein expression levels. However, the spatial localization of the tin RNA and protein were not significantly affected. They then focused on the functional consequences of the tin DPE mutation and found normal dorsal vessel formation, via staining of pericardial cells with Eve, Odd, and tin. In addition, they found significantly decreased viability in mutants with a single copy of DPE mutation in tin and poor heart physiology in DPE-mutant homozygotes. Next, they analyzed the muscle and heart transcriptome of mutant strains and found differentially expressed genes associated with heart, muscle, and nervous system as the most affected. These findings would account for the decreased viability and affected heart function observed in the mutant strains.

These studies are valuable because they highlight the functional importance of the DPE core promoter motif on the regulation of tin, which therefore impacts this developmental protein's ability to interact in developmental gene regulatory networks. The findings are also significant given that a mammalian version of tin (nkx2-5) is necessary for heart formation and also has a DPE in its promoter. The experiments performed in this study outline a path that investigations into core promoter elements of other developmental genes can follow.

Some general concerns with the manuscript relate to consistency across the different experiments presented. For example, the authors show that heart patterning is largely normal in the mDPE homozygotes, but do not show mDPE/null animals, which are shown elsewhere to have reduced viability. Since tin is required for somatic and splanchnic muscle formation, mDPE/null embryos should be analyzed for defects in the three muscle lineages, which should provide some more striking differences from controls than is seen with the existing images, and would provide some explanation for the reduced viability. Similarly, what do the adult hearts look like in mDPE homozygotes and mDPE/null files? It would be important to correlate changes in heart physiology with any changes in heart structure.

In addition, the authors analyze the expression of some cardiac markers in Figure 3, and show some differences in the mutants from controls, but it is not clear if these genes belong to any of the differentially expressed clusters shown in Figure 6.

Additional concerns:
1. Throughout the body of the text and methodology, some of the citations have double parenthesis where there should only be one.

2. Revise for misspelled words throughout the paper.

3. In some figures, the wild type is labeled as “Cas9 (WT)” while in others figures it is labeled just “Cas9.” Additionally, F3 and M6 are only sometimes labeled with “(mDPE).”

4. Figure 1. The sequence immediately following the DPE motif, GGTCGA, is a similar sequence to another downstream promoter element. It would be beneficial for the authors to discuss if there is any endogenous promoter activity as there could be an alternative DPE the transcription machinery is utilizing.

5. Figure 2. The protein levels in the Western Blots were analyzed using ImageJ, but the exact numerical quantification data is not included. It is not stated whether the two-tailed Student t-test is unpaired or paired.

6. For the RT-qPCR, the authors write in the methods that “Each sample was run in triplicates.” It is unclear if this was three different biological samples that were run, or if it was the same biological sample run three times, and should be explicitly stated. Furthermore, while the RT-qPCR data suggests a significant increase in Mef2 and Eve in mutant embryos compared to Cas9, the

© 2024. Published by The Company of Biologists under the terms of the Creative Commons Attribution License (https://creativecommons.org/licenses/by/4.0/).

images in Panels B and C do not reflect those results. Can the authors speculate on why this might be? Additionally, it would be good to see immunostained images representing the significant decrease and patterning expression of Doc2 and svp as well.

7. Figure 4. On the bottom row of the figure where it lists the non-Sb:Sb ratio, it should be noted that it is the approximate ratio, as the data in the graph above shows differently. Why is the value >1.0 in the control crosses? Furthermore, it would be beneficial to use the same units in the ratios listed at both the top and bottom. The top ratio is listed in decimal form, while the bottom is listed as a ratio. Tinman is also required for visceral muscle specification and some skeletal muscle formation during development. It might be worth mentioning that reduced viability could also be due to a decrease in the ability of the flies to move.

8. For Figure 6 it would be helpful to label the numerical column in Figure A. Also, it is not clear how clusters #920, #193, #19, and #977 were chosen amongst all the clusters. Were these the only clusters enriched for the aforementioned GO terms? What was the total number of clusters analyzed? It would be helpful to further explain these choices.

Comments for the author

Journal: Development
MS ID#: DEVELOP/2023/202355
From promoter motif to cardiac function: A single DPE motif affects transcription regulation and organ function in vivo

The manuscript by Sloutskin et al. uses Drosophila melanogaster to study the effects of mutating the downstream core promoter element (DPE) of the endogenous tin gene. The authors used CRISPR to create two independent mutant strains and found reduced tin RNA and protein expression levels. However, the spatial localization of the tin RNA and protein were not significantly affected. They then focused on the functional consequences of the tin DPE mutation and found normal dorsal vessel formation, via staining of pericardial cells with Eve, Odd, and tin. In addition, they found significantly decreased viability in mutants with a single copy of DPE mutation in tin and poor heart physiology in DPE-mutant homozygotes. Next, they analyzed the muscle and heart transcriptome of mutant strains and found differentially expressed genes associated with heart, muscle, and nervous system as the most affected. These findings would account for the decreased viability and affected heart function observed in the mutant strains.

These studies are valuable because they highlight the functional importance of the DPE core promoter motif on the regulation of tin, which therefore impacts this developmental protein's ability to interact in developmental gene regulatory networks. The findings are also significant given that a mammalian version of tin (nkx2-5) is necessary for heart formation and also has a DPE in its promoter. The experiments performed in this study outline a path that investigations into core promoter elements of other developmental genes can follow.

Reviewer 2

Advance summary and potential significance to field

Slutskin et al., in their manuscript analyse the role of distal promoter element (DPE) upstream of tin coding sequence. To do so they create tinDPE mutants. They measure tin transcript and protein levels during embryonic development and show that DPE has an important role in maintaining the level of tin transcription at early development until 6h AEL while after that, in later embryonic stages tin transcripts level in DPE devoid tin mutants remains normal. There is no impact on both early and late embryonic Tin expression pattern. The influence of DPE on tin transcription seems only partially reproduced at Tin protein level, however presented in main and sup figures western blot analyses differ between samples of the same developmental time window and do not always illustrate author's comments and quantifications (see specific comments below). In a similar way, whole embryo staining for different Tin targets are difficult for me to interpret considering that I am unable to refer with confidence to modification of Tin protein level at a given developmental time point. Presented analyses of heart function suggest that DPE element is important for tin

© 2024. Published by The Company of Biologists under the terms of the Creative Commons Attribution License (https://creativecommons.org/licenses/by/4.0/).

expression regulation in the adult heart. It would be thus important to provide evidence that tin transcript and Tin protein expression is modified in tinDPE adult flies.
csRNAseq performed in tinDPE embryos versus wt embryos at 4 time points allowed to identify differentially expressed genes whose nascent expression shows particular developmental patterns. However, authors provide only a general view on these interesting transcriptional datasets. Showing how behave in this transcriptional analyses known Tin targets including Eve and Mef2 (shown by authors as up regulated in tinDPE embryos) and Doc and Svp (that were shown to be down regulated) will be of interest and would provide an additional information on nascent transcription of those Tin targets.
Thus, present manuscript version appears too preliminary for publication in Development.

Comments for the author

Specific comments:

Western blot presented in Fig. 2A seems not to be representative of quantifications of Tin protein levels below in Fig. 2B. From WB of 8-10h AEL one would predict that Tin protein level in tinDPE versus wt is much more than two times lower.

Western blots in Fig. S2 do not support western blots in Fig. 2A especially for 8-10h AEL.

On the other hand reduction of Tin protein levels in tinDPE context at 8-10h AEL is surprising considering that at RNA level there is no difference with WT and knowing that in mid-stage embryos Tin expression is restricted to the developing heart.

Fig. S2 – authors say « overall Tinman levels are markedly reduced at 2-4h…. »
Tin-specific band in the western blot at this time window is difficult to distinguish in all three conditions – either figure or author's comments appear inappropriate.

Fig. S2 - in figure legend authors say « membrane was first blotted with rabbit α-Tinman antibodies » - Please rephrase

The same data are presented for Tin protein in Fig. 2B and Fig. 3A

Fig. S3 is dispensable - showing that in tin null mutants there is no heart staining whereas in heterozygous tin embryos heart development markers are expressed is known for more than 30 years….
Fig. S4 – authors present Odd staining in tinDPE context in which Odd pericardial cells are not clearly detected. They propose that this is due to ectopic Odd expression that masks pericardial cells. It could be also due to a poor quality of Odd staining as epidermal Odd stripes are not seen either.

Reviewer 3

Advance summary and potential significance to field

Sloutskin et al. generated CRISPR-induced point mutations in a core promoter element called DPE in the Drosophila tinman gene and performed a thorough analysis of how gene expression and the phenotype of these Drosophila embryos are changed.
They observe lowered expression of tinman and some of the downstream target genes as well as an altered physiology of the heart.

Overall, this is a straightforward paper and a nice demonstration of how the function of a core promoter element is required in vivo. The analysis is thorough and makes sense.

Comments for the author

The only question that the authors did not address is whether the DPE plays a role in Pol II pausing. There is generally a correlation between the presence of the DPE and Pol II pausing, but it is not

© 2024. Published by The Company of Biologists under the terms of the Creative Commons Attribution License (https://creativecommons.org/licenses/by/4.0/).

clear whether there is a direct causative relationship. Therefore, the presented data seem like a good opportunity to address this question. The authors generated data for nascent RNA, which one would think would provide a measurement of the nascent transcripts that come from pausing. But this depends on how the library was made and which size selection took place. Have the authors looked into this question?

First revision

Author response to reviewers' comments

We thank the reviewers for their valuable comments and insightful suggestions that helped us improve the manuscript. We have performed multiple experiments and analyses to address the reviewers' comments using different methodologies. These include:

1. Western blot analyses were performed using protein extracts purified from embryos collected on 4-6 different occasions (biological replicates). Each biological replicate included the collection of embryos at the following time intervals: 2-4h, 4-6h, 6-8h (a time interval that was not analyzed in the original manuscript), 8-10h and 10-12h. The revised manuscript includes the exact numerical quantification data for the protein levels analyzed (Fig. S2 and Supplemental File S1). We conclude that Tinman protein levels are significantly lower in 4–6h *tin*^{mDPE} embryos compared to *tin*^{WT}.

2. Immunostaining of homozygous *tin*^{WT} and *tin*^{mDPE} embryos with the following antibodies:

- anti-Svp antibodies (Fig. 2B)
- anti-Doc2 antibodies (Fig. 2C).

The new results support our conclusion that although the expression of distinct Tinman target genes is affected in early *tin*^{mDPE} embryos, dorsal vessel formation, as evident from Svp, Doc2, Mef2 and Eve protein expression patterns at later stages appears intact in *tin*^{mDPE} embryos.

3. Immunostaining of embryos resulting from crosses of homozygous *tin*^{WT} or *tin*^{mDPE} with *tin*^{346}/[TM3, *eve*-LacZ] using the following antibodies:

- anti-Tinman antibodies (Fig. 4A).
- anti-Svp antibodies (Fig. 4B).
- anti-B3Tubulin antibodies (to visualize somatic muscles; Fig. S5A).
- anti-Org1 antibodies (to visualize visceral muscles; Fig. S5B).
- anti-Mef2 antibodies (to examine changes in its expression in cardioblasts; Fig. 5).

Reduced numbers of Tinman-positive cardioblasts (Fig. 4A), Svp-positive cardioblasts (Fig. 4B), and Mef2-positive cardioblasts (Fig. 5) are detected in *tin*^{mDPE} embryos when tested *in trans* to the *tin* null mutation, indicating that the reduced *tin* activity in this genetic background leads to the specification of fewer cardioblasts. Notably, although Tin is required for somatic and visceral muscle formation, dorsal somatic musculature and visceral musculature are unaffected as predicted by the staining patterns of β3Tubulin and Org1, respectively (Fig. S5). Thus, heart development seems to be more dosage sensitive with respect to *tin* activity as compared to somatic musculature and visceral musculature.

4. Immunostaining of dissected and fixed adult hearts to determine cardiac diameters in both male and female flies (Fig. 7A)

Measurements of the diameters of fixed hearts in both 21-day old female and male flies revealed significantly reduced end diastolic diameter values in both *tin*^{mDPE} strains (F3 and M6) compared to *tin*^{WT} strain (Fig. 7A), confirming the *in vivo* data.

© 2024. Published by The Company of Biologists under the terms of the Creative Commons Attribution License (https://creativecommons.org/licenses/by/4.0/).

5. We used the FlyBowl system to measure the locomotor activity features of 21-day-old (Fig. 7B,C), 9-day-old (Fig. S6C,D) and 4-day-old (Fig. S6A,B) *tin*^{WT} and *tin*^{mDPE} lines of both male and female flies.

Remarkably, 21-day-old *tin*^{mDPE} fly lines both have significantly reduced walking velocity and average percentage jumping activity, suggesting that the reduced viability of adult flies (Fig. 3) (as well as the reduced locomotor activity; Fig. 7B,C) may result from the impaired heart function.

6. Added the analysis of *tin* and its targets *svp*, *Doc2*, *Mef2* and *eve* nascent transcripts at 0-2h, 2-4, 4-6h and 6-8h during embryonic development (Fig. S8).

Nascent RNA counts at 4-6h of *tin* and its target genes *Doc2*, *Mef2* and *eve* (but not *svp*) are similar to their expression levels at 4-6h analyzed by RT-qPCR (Fig. S8).

7. Analysis of core promoter composition of Tin-bound genes previously identified by ChIP analysis (PMID 23326246) using our enhanced ElemeNT2023 tool (PMID 38407414), demonstrating the significant enrichment of DPE motifs in Tin-targets compared to developmental genes (Fig. S14).

The new analyses are presented in new Fig. 1, 2, 4, 5, 7, Supplemental Figures S2, S5, S6, S8, S14 and Supplemental File S1, and the relevant text was modified accordingly.

Reviewer 1:
“These studies are valuable because they highlight the functional importance of the DPE core promoter motif on the regulation of tin, which therefore impacts this developmental protein’s ability to interact in developmental gene regulatory networks. The findings are also significant given that a mammalian version of tin (nkx2-5) is necessary for heart formation and also has a DPE in its promoter. The experiments performed in this study outline a path that investigations into core promoter elements of other developmental genes can follow.”

Thank you.

“The authors show that heart patterning is largely normal in the mDPE homozygotes, but do not show mDPE/null animals, which are shown elsewhere to have reduced viability. Since tin is required for somatic and splanchnic muscle formation, mDPE/null embryos should be analyzed for defects in the three muscle lineages, which should provide some more striking differences from controls than is seen with the existing images, and would provide some explanation for the reduced viability.”

We appreciate the reviewer's insightful suggestion to analyze the *tin*^{mDPE}/*tin*^{null} embryos for potential defects in the three muscle lineages. To address this, we have performed additional immunostaining experiments to examine cardioblasts, as well as somatic and visceral muscles in *tin*^{mDPE}/*tin*^{null} embryos. Specifically, we immunostained embryos resulting from crosses of homozygous *tin*^{WT} or *tin*^{mDPE} with *tin*^{346}/[TM3, *eve*-LacZ] using the following antibodies:

- anti-Tinman antibodies (Fig. 4A).
- anti-Svp antibodies (Fig. 4B).
- anti-ß3Tubulin antibodies (to visualize somatic muscles; Fig. S5A).
- anti-Org1 antibodies (to visualize visceral muscles; Fig. S5B).
- anti-Mef2 antibodies (to examine changes in its expression in cardioblasts; Fig. 5).

Remarkably, the expression pattern of Tin shows frequent gaps in cardioblast rows in *tin*^{mDPE} embryos when tested *in trans* to the *tin* null mutation (Fig. 4A). Notably, Svp expression pattern seems to differ between *tin*^{WT}/*tin*^{346} and *tin*^{mDPE}/*tin*^{346} embryos, with some segments in *tin*^{mDPE}/*tin*^{346} having only one of two Svp-positive cells (Fig. 4B).

Both somatic musculature (detected using anti-β3Tubulin antibodies) and visceral mesoderm (detected using anti-Org1 antibodies) are similar between *tin*^{WT}/*tin*^{346} and *tin*^{mDPE}/*tin*^{346} embryos

© 2024. Published by The Company of Biologists under the terms of the Creative Commons Attribution License (https://creativecommons.org/licenses/by/4.0/).

(Fig. S5), indicating that the reduced viability of *tin*^{mDPE}/*tin*^{346} (Fig. 3) does not result from impaired somatic and/or visceral muscle function.

Remarkably, the number of Mef2-positive cardioblasts is significantly reduced in *tin*^{mDPE} embryos when tested *in trans* to the *tin* null mutation (Fig. 5).

"Similarly, what do the adult hearts look like in mDPE homozygotes and mDPE/null files? It would be important to correlate changes in heart physiology with any changes in heart structure."

Remarkably, while changes in the expression patterns of Tin and its targets Svp and Mef2 are only detected in *tin*^{mDPE}/*tin*^{346}, changes in heart structure are detected in the homozygous *tin*^{mDPE}, even without the background of a null *tin* allele. Measurements of the diameters of fixed hearts in both 21-day old female and male flies revealed significantly reduced end diastolic diameter (EDD) values in both *tin*^{mDPE} strains (F3 and M6) compared to *tin*^{WT} strain (Cas9), confirming the *in vivo* data (Fig. 7A).

"In addition, the authors analyze the expression of some cardiac markers in Figure 3 and show some differences in the mutants from controls, but it is not clear if these genes belong to any of the differentially expressed clusters shown in Figure 6."

NOTE: We have removed unpublished data provided for the referees in confidence.

HOMER algorithm was used for unsupervised hierarchical clustering learning approach, in which similar genes or loci get grouped based on their pattern.
tin and *Doc2* belong to cluster 18 (below), which was not featured in the original Fig. 6 (now Fig. 8), while *svp*, *Mef2* and *eve* do not belong to specific clusters.

Notably, nascent RNA (csRNA-seq) counts at 4-6h of *tin* and its target genes *Doc2*, *Mef2* and *eve* (but not *svp*) are similar to their expression levels at 4-6h analyzed by RT-qPCR (Fig. S8). This is in line with the different nature of these methods, *i.e.*, nascent RNA transcription vs. mature/spliced RNA transcripts.

Additional concerns:

1. Throughout the body of the text and methodology, some of the citations have double parenthesis where there should only be one.

We thank the reviewer for pointing it out. In the revised manuscript, these are the cases with double parenthesis:

- ... using semi-automated heart analysis (SOHA, (Fink et al., 2009)).
- ...(reviewed in (Bodmer and Venkatesh, 1998; Cripps and Olson, 2002; Rotstein and Paululat, 2016))
- ...In parallel, preferred downstream positions required for proper transcriptional output were identified (PDP, (Dreos et al., 2021)).
- ...followed by rabbit anti-Tinman polyclonal antibodies (1:1000 in 3% BSA, (Yin et al., 1997))
- ... HOMER csRNAseq module (http://homer.ucsd.edu/homer/ngs/csRNAseq/index.html, (Duttke et al., 2019))

2. Revise for misspelled words throughout the paper.

The revised manuscript has been revised by a professional English editor, using US English, as permitted by the journal.

3. In some figures, the wild type is labeled as "Cas9 (WT)" while in others figures it is labeled just "Cas9." Additionally, F3 and M6 are only sometimes labeled with "(mDPE)."

We thank the reviewer for drawing our attention to it. To address this issue, we have thoroughly reviewed all figures and corrected the labels to ensure consistency across the manuscript and figures. Specifically:

- All instances of the wild type are now consistently labeled: "*tin*^{WT}."

© 2024. Published by The Company of Biologists under the terms of the Creative Commons Attribution License (https://creativecommons.org/licenses/by/4.0/).

- The labels for the *tin* mutant DPE F3 and M6 lines are now consistently labeled: "*tin*^{mDPE} (F3 or M6)".

4. Figure 1. The sequence immediately following the DPE motif, GGTCGA, is a similar sequence to another downstream promoter element. It would be beneficial for the authors to discuss if there is any endogenous promoter activity as there could be an alternative DPE the transcription machinery is utilizing.

Thank you for bringing this up.

The revised manuscript now includes the following sentence: "Interestingly, csRNA-seq analysis identified that transcription of *tin* in both the *tin*^{WT} and *tin*^{mDPE} lines initiates in a single TSS (Fig. 8A, Fig. S7), which is the same TSS depicted in Fig. 1A."

These experimental findings show that transcription does not initiate from a potential Inr+DPE motif that the reviewer referred to.

5. Figure 2. The protein levels in the Western Blots were analyzed using ImageJ, but the exact numerical quantification data is not included. It is not stated whether the two-tailed Student t-test is unpaired or paired.

We have performed additional western blot analyses using protein extracts purified from embryos collected on 4-6 different occasions (biological replicates). Each biological replicate included the collection of embryos at the following time intervals: 2-4h, 4-6h, 6-8h (a time interval that was not analyzed in the original manuscript), 8-10h and 10-12h.

- We have included the exact numerical quantification data for the protein levels analyzed in the western blots (revised Fig. S2 and Supplemental File S1).
- We have clarified in both the figure legend and the Materials and Methods section (western blot analysis) that the two-tailed t-test used for statistical analysis is unpaired.

6. For the RT-qPCR, the authors write in the methods that "Each sample was run in triplicates." It is unclear if this was three different biological samples that were run, or if it was the same biological sample run three times, and should be explicitly stated.

We have performed >3 separate experiments using embryos collected from different parental populations on different weeks (*i.e.*, biological replicates). Each sample subjected to RT- qPCR was analyzed in 3 technical replicates. We have revised the text to explicitly state the nature of the replicates. Specifically, we have added the following clarification to the legend of Figure 2: "Each sample was run in triplicates (n= biological replicates, each tested in three technical replicates)."

Furthermore, while the RT-qPCR data suggests a significant increase in Mef2 and Eve in mutant embryos compared to Cas9, the images in Panels B and C do not reflect those results. Can the authors speculate on why this might be?

Panel A presents the analysis of RNA expression using RT-qPCR in 4-6h embryos (stages 9-10), whereas panels B-E (including the new staining analyses using anti-Tin, anti-Doc2 and anti-Svp antibodies) present the protein expression patterns of Tin, Svp, Doc2, Mef2 and Eve at later developmental stages (13-17). The differences pointed out by the reviewer result from differences between developmental stages, as well as differences between RNA expression levels and protein expression patterns (which may result from post-transcriptional and post-translational regulation).

Additionally, it would be good to see immunostained images representing the significant decrease and patterning expression of Doc2 and svp as well.

We appreciate the reviewer's suggestion. The revised manuscript includes immunostaining images of embryos using anti-Doc2 and anti-Svp antibodies in both the *tin*^{mDPE} homozygous embryos (as discussed above).

© 2024. Published by The Company of Biologists under the terms of the Creative Commons Attribution License (https://creativecommons.org/licenses/by/4.0/).

The results from these experiments have been included in the revised manuscript, and are now presented in Figure 2 panels B (Svp) and C (Doc2). Interestingly, similarly to Mef2 and Eve protein expression patterns, Svp and Doc2 staining patterns are not affected in homozygous *tin*^{mDPE} embryos compared to homozygous *tin*^{WT} embryos.

7. Figure 4. On the bottom row of the figure where it lists the non-Sb:Sb ratio, it should be noted that it is the approximate ratio, as the data in the graph above shows differently.

We have added the following clarification directly to the figure: "**Expected** non-Sb:Sb ratio" to indicate that the values listed are approximate.

Why is the value >1.0 in the control crosses?

The value of >1.0 ratio in the control crosses of homozygous *tin*^{WT} flies to *tin*^{346}/[TM3, Sb] may result from the use of the TM3 balancer, which is known to confer a disadvantage in terms of viability.

Furthermore, it would be beneficial to use the same units in the ratios listed at both the top and bottom. The top ratio is listed in decimal form, while the bottom is listed as a ratio.

We considered several options for changing this, but using the expected non-Sb:Sb ratios that represent the theoretical values, makes it easier for the reader to understand the genetic crosses and the results. As the measured viability ratios reflect the actual numbers of flies, we present them with a decimal point, rather than as a fraction.

Tinman is also required for visceral muscle specification and some skeletal muscle formation during development. It might be worth mentioning that reduced viability could also be due to a decrease in the ability of the flies to move.

We thank the reviewer for bringing up this important issue. To address it, we have taken two approaches:
a) Immunostaining of *tin*^{WT}/*tin*^{346} and *tin*^{mDPE}/*tin*^{346} embryos using anti-ß3Tubulin antibodies (to visualize somatic muscles; Fig. S5A) and anti-Org1 antibodies (to visualize visceral muscles; Fig. S5B). Notably, both somatic musculature and visceral mesoderm are similar between *tin*^{WT}/*tin*^{346} and *tin*^{mDPE}/*tin*^{346} embryos, indicating that the reduced viability of *tin*^{mDPE}/*tin*^{346} (Fig. 3) does not result from impaired somatic and/or visceral muscle function.

b) Performed FlyBowl analyses to examine whether adult flies present differences in distinct locomotor activity features. Remarkably, significantly reduced walking velocity (velmag) and average percentage jumping activity were observed in both 21-day-old adult male and female *tin*^{mDPE} compared to *tin*^{WT} flies (Fig. 7B,C). No significant changes in the average percentage of walking and turning activities or the turning velocity were observed between *tin*^{WT} and both *tin*^{mDPE} lines (Fig. 7B,C). In 4-day-old and 9-day-old male and female flies, the average percentage jumping activity was lower in both *tin*^{mDPE} lines compared to the *tin*^{WT} line (Fig. S6). Jumping activity declines with age, with a more pronounced reduction observed in *tin*^{WT} flies. These findings suggest that impaired heart function, reduced walking velocity and decreased average percentage jumping activity may contribute to the reduced viability (Fig. 3).

8. For Figure 6 it would be helpful to label the numerical column in Figure A.
As suggested, we have labeled the numerical column: "Reads", representing the counts in each fly line: *tin*^{WT} (Cas9), *tin*^{mDPE} (F3, or M6) in each time interval.

Also, it is not clear how clusters #920, #193, #19, and #977 were chosen amongst all the clusters. Were these the only clusters enriched for the aforementioned GO terms? What was the total number of clusters analyzed? It would be helpful to further explain these choices.

All the resulting clusters are depicted in Fig. S10 and fully described in File S3. We used HOMER unsupervised hierarchical clustering learning approach, in which similar genes or loci get grouped based on their pattern. The output of the analysis was 14 HOMER-validated clusters. Of these, four clusters (#920, #193, #19, and #977) were selected for further discussion due to their notable

© 2024. Published by The Company of Biologists under the terms of the Creative Commons Attribution License (https://creativecommons.org/licenses/by/4.0/).

expression patterns. The selected GO terms offer a snapshot of the key features of each cluster. The expression patterns and a comprehensive list of GO terms associated with each of the 14 clusters are detailed in File S2.

Reviewer 2:

The influence of DPE on tin transcription seems only partially reproduced at Tin protein level, however presented in main and sup figures western blot analyses differ between samples of the same developmental time window and do not always illustrate author's comments and quantifications (see specific comments below). In a similar way, whole embryo staining for different Tin targets are difficult for me to interpret considering that I am unable to refer with confidence to modification of Tin protein level at a given developmental time point.

We apologize. To address this comment, we have performed additional western blot analyses using protein extracts purified from embryos collected on 4-6 different occasions (biological replicates). Each biological replicate included the collection of embryos at the following time intervals: 2-4h, 4-6h, 6-8h (a time interval that was not analyzed in the original manuscript), 8-10h and 10-12h. We have included the exact numerical quantification data for the protein levels analyzed in the Western Blots. This data is now presented in the revised Fig. S2 and Supplemental File S1.

We conclude that Tinman protein levels are significantly lower in 4–6h *tin*^{mDPE} embryos compared to *tin*^{WT}.

Presented analyses of heart function suggest that DPE element is important for tin expression regulation in the adult heart. It would be thus important to provide evidence that tin transcript and Tin protein expression is modified in tinDPE adult flies.

Thank you. We agree that the analyses of the levels of both *tin* transcript and Tin protein in the adult would be interesting. The revised manuscript includes the analysis of both adult heart diameter measurements and adult fly activity features, in addition to adult heart physiology. Moreover, we have recently tested whether *tin*^{mDPE} affects pupae viability when tested *in trans* to the *tin* null allele. To this end we used the TM6B balancer chromosome, which carries the Tubby (Tb) allele, and scored the ratio of non-Tb to Tb pupae resulting from the crosses of either *tin*^{WT} or *tin*^{mDPE} with *tin*^{346}/TM6B flies.

NOTE: We have removed unpublished data provided for the referees in confidence.

Remarkably, we observed that *tin*^{mDPE} affects pupae viability when tested *in trans* to the *tin* null allele. Thus, we would like to expand this preliminary analysis and include the analysis of adult *tin* RNA and Tin protein levels in a future project. We believe such analyses are beyond the scope of this manuscript.

csRNAseq performed in tinDPE embryos versus wt embryos at 4 time points allowed to identify differentially expressed genes whose nascent expression shows particular developmental patterns. However, authors provide only a general view on these interesting transcriptional datasets. Showing how behave in this transcriptional analyses known Tin targets including Eve and Mef2 (shown by authors as up regulated in tinDPE embryos) and Doc and Svp (that were shown to be down regulated) will be of interest and would provide an additional information on nascent transcription of those Tin targets.

We thank the reviewer for bringing it up. Fig. S8 of the revised manuscript depicts csRNA counts of *tin* and its target genes *svp*, *Doc2*, *Mef2* and *eve*. Notably, nascent RNA counts at 4-6h of *tin* and its target genes *Doc2*, *Mef2* and *eve* (but not *svp*) (Fig. S8) are similar to their expression levels at 4-6h analyzed by RT-qPCR (Fig. 2A). This is in line with the different nature of these methods, *i.e.*, analysis of transcription initiation (csRNA-seq) vs. expression levels (RT-qPCR).

Moreover, raw csRNA-seq data is accessible at GSE221852, with complete cluster analysis and expression data provided in Files S2 and S3 for public use.

Specific comments:

© 2024. Published by The Company of Biologists under the terms of the Creative Commons Attribution License (https://creativecommons.org/licenses/by/4.0/).

Western blot presented in Fig. 2A seems not to be representative of quantifications of Tin protein levels below in Fig. 2B. From WB of 8-10h AEL one would predict that Tin protein level in tinDPE versus wt is much more than two times lower.

As detailed above, we have performed additional western blot analyses using protein extracts purified from embryos collected on 4-6 different occasions (biological replicates). Each biological replicate included the collection of embryos at the following time intervals: 2-4h, 4-6h, 6-8h (a time interval that was not analyzed in the original manuscript), 8-10h and 10-12h. We have included the exact numerical quantification data for the protein levels analyzed in the western blots (revised Fig. S2 and Supplemental File S1).

Western blots in Fig. S2 do not support western blots in Fig. 2A especially for 8-10h AEL. On the other hand reduction of Tin protein levels in tinDPE context at 8-10h AEL is surprising considering that at RNA level there is no difference with WT and knowing that in mid-stage embryos Tin expression is restricted to the developing heart.

As detailed above, we have performed additional western blot analyses, and modified the text accordingly. Due to large variations in protein expression, we have performed these experiments using embryos collected on 4-6 separate occasions (biological replicates). Whereas the levels of *tin* RNA are significantly lower in 4-6h tin^{mDPE} embryos compared to tin^{WT} (Fig. 1B, Fig. 2A), the levels of *tin* RNA are similar between tin^{WT} and tin^{mDPE} in 6-7h and 7-8h embryos (Fig. 1B), in line with Tin protein expression levels (Fig. S2, Supplemental File S1).

Fig. S2- authors say « overall Tinman levels are markedly reduced at 2-4h…. » Tin-specific band in the western blot at this time window is difficult to distinguish in all three conditions – either figure or author's comments appear inappropriate.

As detailed above, we have performed additional western blot analyses, and modified the text accordingly.

Fig. S2- in figure legend authors say « membrane was first blotted with rabbit α-Tinman antibodies » - Please rephrase

The legend was rephrased: "Western blotting of each membrane was initially performed using rabbit anti-Tinman antibodies. The levels of Actin as a loading control were detected using mouse anti-Actin antibodies."

Moreover, the relevant paragraph in the Materials and Methods section was rephrased:

"Western blot analysis

Protein extracts from 2-4h, 4-6h, 6-8h, 8-10h and 10-12h dechorionated embryos were prepared in 2X DTT-based sample buffer at a final concentration of ~0.5mg embryos/µl. 15-20µl of the sample was analyzed using 10% SDS-PAGE gel, followed by rabbit anti-Tinman polyclonal antibodies (1:1000 in 3% BSA, (Yin et al., 1997)) and then by goat-anti-rabbit IgG- HRP (1:5000 in 5% milk, Jackson ImmunoResearch). HRP signal was detected using EZ- ECL kit (Biological Industries) or Luminata Crescendo Western HRP substrate (MERCURY), and imaged using iBright Imaging System (Thermo). The use of the Tinman antibody results in background bands, however the major band is above the 45kDa size marker, as predicted. The same membrane was stripped (ST010, Gene Bio-Application) and re-blotted with mouse anti-Actin monoclonal antibodies (1:1000 in 3% BSA, Abcam ab8227) to ensure proper gel loading. Images were quantified using the iBright Analysis Software (Thermo); each sample was normalized to the detected Actin levels. Statistics was calculated with unpaired two-tailed one-sample t-test followed by Bonferroni correction for multiple testing."

The same data are presented for Tin protein in Fig. 2B and Fig. 3A.

We apologize for the misunderstanding. The revised text specifically indicates that protein expression levels are shown in Fig. 2B (now 1C), and RNA expression levels are shown in Fig. 3A (now 2A).

© 2024. Published by The Company of Biologists under the terms of the Creative Commons Attribution License (https://creativecommons.org/licenses/by/4.0/).

Fig. S3 is dispensable - showing that in tin null mutants there is no heart staining whereas in heterozygous tin embryos heart development markers are expressed is known for more than 30 years….

This figure was removed from the manuscript, as requested.

Fig. S4- authors present Odd staining in tinDPE context in which Odd pericardial cells are not clearly detected. They propose that this is due to ectopic Odd expression that masks pericardial cells. It could be also due to a poor quality of Odd staining as epidermal Odd stripes are not seen either.

We agree that the Odd staining pattern (now shown in Fig. S3) could perhaps result from the quality of the antibodies. To better visualize the dorsal vessel, we performed additional immunostaining experiments using anti-Svp and anti-Doc2 antibodies (Fig. 2B,C). Our findings are summarized in the following sentence in the manuscript: "…, based on Tin and Odd staining patterns (Fig. S3), as well as on Svp, Doc2, Mef2 and Eve staining patterns (Fig. 2B-E), proper dorsal vessel formation is evident in both homozygous tin^{mDPE} embryos."

Reviewer 3:

Overall, this is a straightforward paper and a nice demonstration of how the function of a core promoter element is required in vivo. The analysis is thorough and makes sense.

Thank you.

The only question that the authors did not address is whether the DPE plays a role in Pol II pausing. There is generally a correlation between the presence of the DPE and Pol II pausing, but it is not clear whether there is a direct causative relationship. Therefore, the presented data seem like a good opportunity to address this question. The authors generated data for nascent RNA, which one would think would provide a measurement of the nascent transcripts that come from pausing. But this depends on how the library was made and which size selection took place. Have the authors looked into this question?

We thank the reviewer for bringing this up. Indeed, previous analyses by Mike Levine's and Julia Zeitlinger's labs have demonstrated the enrichment of DPE motifs in Pol II paused promoters. Sequence examination of the *tin* promoter predicts that it includes the *Drosophila* pause button (PB) motif (which was found to be enriched in *Drosophila* paused promoters) at +32-38 relative to the TSS. As the size selection for the csRNA-seq analysis was 20-60 nucleotides, we cannot unequivocally determine the pausing state of the promoter.

Second decision letter

MS ID#: DEVELOP/2023/202355

MS TITLE: From promoter motif to cardiac function: A single DPE motif affects transcription regulation and organ function in vivo

AUTHORS: Anna Sloutskin, Dekel Itzhak, Georg Vogler, Hadar Pozeilov, Diana Ideses, Hadar Alter, Orit Adato, Hadar Shachar, Tirza Doniger, Galit Shohat-Ophir, Manfred Frasch, Rolf Bodmer, Sascha H Duttke, and Tamar Juven-Gershon

I am happy to tell you that your manuscript has been accepted for publication in Development, pending our standard publication integrity checks.

Reviewer 1

© 2024. Published by The Company of Biologists under the terms of the Creative Commons Attribution License (https://creativecommons.org/licenses/by/4.0/).

Advance summary and potential significance to field

The authors have done a good job of responding to my comments.

Comments for the author

The authors have done a good job of responding to my comments.

Reviewer 3

Advance summary and potential significance to field

The authors have thoroughly addressed the reviewers' concerns in the revised version.

Comments for the author

The authors have thoroughly addressed the reviewers' concerns in the revised version.

© 2024. Published by The Company of Biologists under the terms of the Creative Commons Attribution License (https://creativecommons.org/licenses/by/4.0/).
